# Reshaping heavy rare earth supply chains amidst China's stringent environmental regulations

**DOI:** 10.1016/j.fmre.2023.11.019

**Published:** 2024-01-26

**Authors:** Wei Chen, Peng Wang, Fanran Meng, Alexandra Pehlken, Qiao-Chu Wang, Wei-Qiang Chen

**Affiliations:** a Department of Environmental Science and Engineering, University of Science and Technology of China, Hefei 230026, China; b State Key Laboratory for Ecological Security of Regions and Cities, Institute of Urban Environment, Chinese Academy of Sciences, Xiamen 361021, China; c University of Chinese Academy of Sciences, Beijing 100049, China; d Ganjiang Innovation Academy, Chinese Academy of Science, Ganzhou 341119, China; e Department of Chemical & Biological Engineering, The University of Sheffield, Sheffield, S1 3JD, United Kingdom; f OFFIS – Institute for Information Technology, 26121 Oldenburg, Germany

**Keywords:** Terbium, Heavy rare earth elements, Supply chain bottlenecks, Green mining innovation, Sustainable transition

## Abstract

China's official heavy rare earths (HREs) supply, vital to the global sustainable transition, has declined by 90% over the past 20 years. Global concerns have mounted regarding China's production quota policies, yet the real-world bottlenecks remain unclear. This study explores China's terbium (a critical HREs element) supply-demand conflicts and supply chain bottlenecks, and further simulates future potential changes. We identify a growing terbium shortage (a total of 3300 metric tons) in China as its registered production declined by 90% during the period from 2007 to 2018. Contrary to previous views that attribute HREs supply limitations to the production quota policy, we find that only 25% of China's quota related to HREs was utilized in 2018. Such a large quota-supply gap stems primarily from the enforced closures of HREs mines since the current mining techniques failed to reach strict environmental regulations. Furthermore, our simulations predict a 2–5-fold increase in terbium shortage by 2060 under the burgeoning ambitions in electric vehicles and wind power. However, this looming shortage could potentially be mitigated by 27%–70% under the scenario of breakthroughs in green mining techniques. This study highlights the urgency of seeking and promoting HREs green mining technologies, with implications for shifting global attention from geopolitical competition to green supply of rare earth and other minerals.

## Graphical abstract

**Figure:**
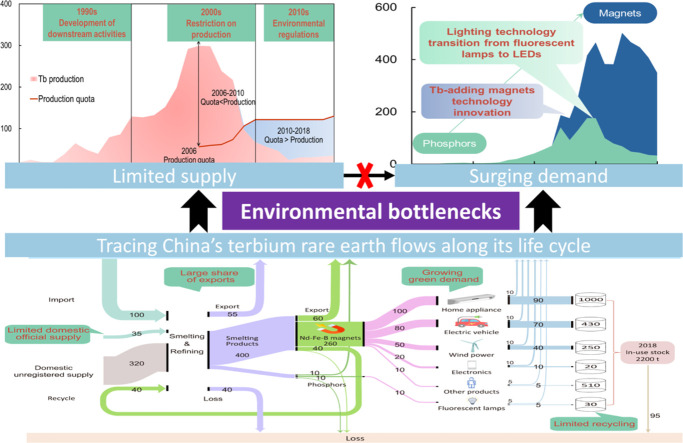

## 1 Introduction

Heavy rare earths (HREs) are essential to a wide range of low-carbon technologies that are indispensable for supporting the global sustainable transition [1]. HREs represent a subset of rare earth elements, comprising gadolinium, terbium, dysprosium, holmium, erbium, thulium, ytterbium, and lutetium. Among them, terbium has gained particular attention due to its increasing use in low-carbon energy technologies such as electric vehicles and wind turbines [2]. Notably, terbium stands out as one of the rarest HREs, with a content of less than 0.01% in most rare earth mineral deposits, often geographically concentrated in a few countries (mainly China) [3]. China has emerged as the world's largest supplier and exporter of HREs since the beginning of the 21st century, raising growing global concerns regarding China's control over HRE supply [4], [5], [6]. Consequently, the U.S. [7], [8], [9], EU [10], [11], [12], Japan [13], and some other nations [14], have deemed terbium as a critical metal, acknowledging the substantial supply risks associated with their heavy dependence on a single major source [15].

Concerns about the concentration of HRE supply in China have largely centered on China's production policy in this regard. After years of excessive exploitation and domestic mining of HREs, in 2006 China enacted the rare earth production quota policy, which dictated the allowable domestic production levels of rare earth concentrates [16]. In 2011, China employed environmental policies and industrial standards specifically tailored to HREs production [17]. These policies and standards gained global attention for their perceived "restrictiveness", raising concerns about China's willingness to HRE supply on an international scale. During the period from 2017 to 2021, the U.S. presidents signed three executive orders aimed at addressing the potential threat posed by foreign adversaries in the realm of critical minerals, with a particular focus on HREs [18], [19], [20]. However, the quantitative understanding of China's HRE supply changes and the driving forces behind these changes has remained sparse, making it challenging to pinpoint the real-world bottlenecks of China's HRE supply chain.

Analyzing the supply chain dynamics is essential for pinpointing bottlenecks [21], particularly when dealing with HREs as strategic resources with small market size, which can be easily influenced by governmental interventions, technological changes, geopolitical relationships, etc. [16,[22], [23], [24]. In this context, some price-driven mechanisms and approaches cannot well capture demand and supply dynamics, particularly in the long-term trend analysis. As highlighted in the U.S. National Science and Technology Council (NSTC) [25], there are two approaches to investigate the supply risks of critical minerals, namely the criticality assessment-based screening approach, and the material flow analysis-based deep dive analysis approach. The criticality assessment model, as proposed by the U.S. National Research Council (NRC) in 2006 [26], has gained widespread adoption by influential entities like the U.S. Department of Energy [27], European Commission [11,^12^,28], Yale University [15], and others [29]. However, criticality assessment lacks a detailed and dynamic supply chain risk analysis [30]. For comprehensive supply chain risk analyses, the Material Flow Analysis method (MFA) stands out as a valuable tool for tracking material flows and stocks, enabling the identification of the supply chain risk and laying the foundation for well-informed decision-making regarding critical metals [21]. MFA has been widely applied in mapping the supply chain risks of various metals, including lithium [31], cobalt [32], nickel [33], indium [34], gallium [34], and others [35]. In addition, MFA has been utilized to map the supply chain movements of europium [36] and dysprosium [37,38] within the HREs industry. Nevertheless, there remains a significant knowledge gap when it comes to quantifying supply bottlenecks for terbium.

In this context, we construct a dynamic material flow analysis (MFA) method, coupled with China's HREs policy, and employ a scenario analysis approach. This integrated methodology is applied to illuminate the complex landscape of terbium supply-demand dynamics in China, taking a comprehensive supply chain perspective that spans from mining, refining, manufacturing, usage, to end-of-life considerations. The primary objective of this study is to explore the real supply chain bottlenecks and propose corresponding measures, while also assessing their potential impact.

## 2 Materials and methods

### 2.1 Supply chain mapping

This study follows the standard dynamic MFA method to trace Tb stocks and flows in China annually from 1990 to 2018. The system definition and framework are illustrated in Fig. S1. The spatial system boundary refers to the geographical border of Chinese mainland (Taiwan, Hongkong, and Macau are excluded). All flows and stocks are expressed in terms of metric ton Tb metallic equivalents.

The life cycle of Tb is divided into six main stages including mining and beneficiation, separation and refining, fabrication, manufacturing, use, and waste management. Unlike most rare earth elements from Bayan Obo ore deposits in northern China, the concentration of Tb is mainly extracted from the ionic rare earth deposits in southern provinces of China (e.g., Jiangxi Province). Notably, the Tb in this concentrate is generally mixed with other HREs and needs to be further separated and refined into Tb primary products (in the form of Tb oxides, Tb metals, Tb chlorides, and others). In fabrication, Tb refined products are converted into intermediate products, mainly rare earth phosphors and Nd-Fe-B permanent magnets. The modern lighting industry is dependent on fluorescent materials such as tricolor phosphors, and Tb oxide is indispensable for emitting green light in tricolor phosphors; Tb is also utilized to enhance Nd-Fe-B permanent magnets properties. Meanwhile, there are always around 20%−30% losses in the production of Nd-Fe-B magnets, which are recycled in the refining process. In the manufacturing stage, Tb-containing phosphors are mainly used to produce fluorescent lamps, flat-panel displays, and cathode-ray tubes. Tb-based Nd-Fe-B magnets are widely used, and the most important applications include air conditioners, mobile phones, electric vehicles, and wind turbines. After manufacturing, Tb enters the use phase in the form of final products, which are further categorized into five end-use sections in this study: home appliances, electronics, transportation, wind turbines, and others. Based on the product lifetime, Tb can remain in society for different periods. In the waste management stage, there is currently little recovery of Tb from end-of-life products. Detailed information about the supply chain analysis was presented in Tables S1-S3.

All calculations are based on the mass balance principle of MFA: the total input is equal to the total output for each process, which is expressed by Eq. 1:

(1) $$F^{Pre}\left(i,t\right)+F^{Import}\left(i,t\right)=F^{Next}\left(i,t\right)+F^{Export}\left(i,t\right)+F^{Loss}\left(i,t\right)$$

where *i* is the index for life process, *t* is the index for the studied year; $F^{Pre}\left(i,t\right)$ equals the amount of Tb contained in the goods from life stage *i* in the year *t*; $F^{Import}\left(i,t\right)$ equals the amount of Tb embodied in imported Tb-containing goods in life stage *i* in the year *t*; $F^{Next}\left(i,t\right)$ equals the amount of Tb contained in the goods to life stage *i* in the year *t*; $F^{Export}\left(i,t\right)\;$ equals the amount of Tb embodied in exported Tb-containing goods in life stage *i* in the year *t*; $F^{Loss}\left(i,t\right)$ equals the loss of Tb contained in the goods in life stage *i* in the year *t*.

Tb in-use stock is the amount of Tb that is still in active use in society. The in-use stock is changing annually and this study used the top-down approach to determine Tb in-use stocks. The basic principle is that in-use stock for the current year is calculated based on the stock for the previous year, as well as the current year's net inflow, as expressed by Eq. 2:

(2) $$S^{in-use}\left(t\right)=S^{in-use}\left(t-1\right)+F^{Net\;input}\left(t\right)$$

where $S^{in-use}\left(t\right)$ and $S^{in-use}\left(t-1\right)$ are the in-use stock at the year *t* and its previous year, respectively; $F^{Net\;inout}\left(t\right)$ means the net input volume of Tb in the use stage of the year *t*.

### 2.2 Rare earth production quota allocation

Notably, China's rare earth production quotas are directly allocated to each province or firm. This study calculated China's Tb production quota by multiplying the rare earth production quotas in each province by their Tb content, as shown in Eq. 3. The information about HREs production quota was presented in Table S4 and Fig. S2.

(3) $$Q^{Tb}\left(t\right)=\sum_{1}^{k}\left(Q_{i,t}\times C_{i}\right)$$

where $Q^{Tb}\left(t\right)$ means China's production quota of Tb in the year of t, $Q_{i,t}$ means the production of Tb in the year of t in i province, $C_{i,t}$ means Tb content of the rare earth mine deposit in *i* province.

### 2.3 Terbium future demand scenarios

This study follows a "climate targets-low carbon applications paths-metal demand" nexus framework to simulate the future Tb demand and sets three scenarios, which are the baseline scenario, the Stated Policies scenario (STEPS), and the Net Zero scenario. The low-carbon applications considered in the sector include electric vehicles, wind power, and home appliances. The baseline scenario indicates that Tb-containing industries follow the historical development pathways. The STEPS scenario shows the low carbon technologies paths under China's nationally determined contributions routes. The Net Zero scenario represents their paths under the net zero emissions ambitions by 2060 in China. The detailed prediction method was shown in the fourth part of the Supporting Information and more results were shown in Figs. S3-S4.

### 2.4 Terbium supply shortage mitigation scenarios

This study sets four Tb supply shortage mitigation scenarios to quantify the future changes of supply shortages. For the first effort, we assume that China can fully utilize its production quota (given that only 25% of the quota was utilized in 2018). In the second scenario, we assume China's quota can be soon relaxed to the highest level in history, which means the production level in 2006. Note that, both approaches can be achieved only if green mining techniques get a breakthrough. The third scenario is that the recycling stream of end-of-life products can be drastically used as feedstock. Furthermore, we established three import scenarios based on historical trade trends: growth, constant, and decline scenario. Under the growth scenario, we projected a 2% growth rate based on historical trends. In the constant scenario, we assumed imports would maintain at the 2018 level. Moreover, we set a decline scenario, with a negative growth rate of 46% based on the trade data from 2020 to 2022.

### 2.5 Data sources and uncertainty analysis

Limitations and uncertainties of our analysis are mainly limited by the various quality of data. Therefore, we apply the Monte Carlo simulation to examine the impacts of all input parameters on our results. Monte Carlo simulation has commonly been used to simulate the uncertainty of MFA results. The uncertainty in parameters (e.g., lifetime, content, product quality) is modeled with a normal distribution with three levels of coefficient of variation (2%, 5%, and 10%). The Monte Carlo simulation was applied 10,000 times, and the results are provided in Fig. S6. Simulation results show that the uncertainties have limited impacts on our results.

## 3 Results

**Fig. 1:**
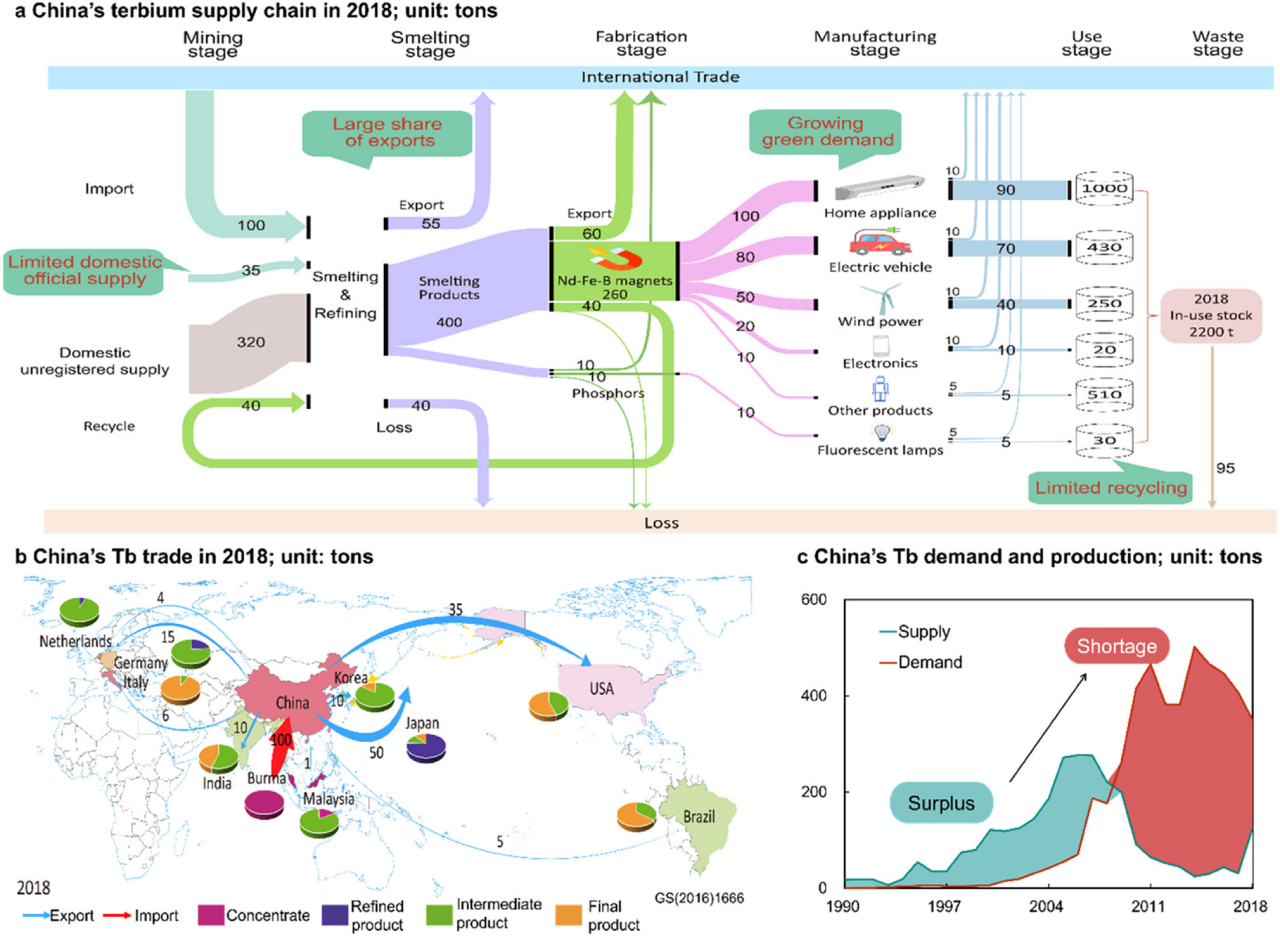
**China's terbium supply chain in 2018.** (a) The flows and stocks of terbium in China: the colors are utilized therein to aid in the life cycle stage, while the width of the flows reveals the magnitude of the flows; (b) China's Tb trade; (c) the conflict between Tb supply and demand in China. All flow values are in tons Tb.

This research offers a comprehensive map of the Tb supply chain in China, encompassing supply, demand, losses, recycling, and trade, with the results for the year 2018 displayed in Fig. 1a (additional results are presented in Figs. S4-S9). The differences between supply and demand are attributed to the unregistered sources, primarily from "illegal" mining activities and potential statistical discrepancies. Regarding recycling, we focus on the recycling of waste flow in processing stage, while the recycling of end-of-life products remains negligible.

### 3.1 China's growing shortage of terbium

In 2018, a total of 175 t (tons, hereafter) of Tb mining feedstocks were fed into smelting and refining facilities in China for formal statistics. These feedstocks were sourced as follows: 20% from official registered production, 57% from import, and 23% from recycling. On the demand side, 340 tons Tb were required to produce Nd-Fe-B magnets, which were further manufactured into home appliances (38%), electric vehicles (30%), wind power (19%), and other products (13%). In addition, a relatively smaller quantity of 20 tons of Tb was needed in phosphors, primarily for fluorescent lamps. In the use stage, a cumulative total of 2200 tons of Tb was stocked in China by the end of 2018. Within the in-use stock, 95 tons of Tb became scrap as products reached the end of their lifetime. Additionally, 40 tons of Tb were recycled from the scrap generated during the Nd-Fe-B processing.

Regarding trade, as a major producer and exporter of HRE, China exported a total of 136 tons of Tb to the rest of the world in 2018 (Fig. 1b). These exports took various product forms, with 34% in smelted products, 36% in fabricated products, and 30% in manufactured products. In terms of importing countries, Japan was the largest importer of Tb from China, accounting for 37% of China's total exports, followed by the U.S. and Europe, which imported 35 and 25 tons of Tb from China, respectively.

Our results reveal a significant shift in China's Tb supply dynamics from surplus to shortage, by comparing the demand for Tb and its formal statistical supply (official registered production, recycling, and import, excluding unregistered production). Fig. 1c illustrates the distinct temporal patterns of China's Tb supply and demand during the study period. Initially, as a constituent of co-mined HREs, Tb production in China surged by 15 times from 1990 to 2006, driven by the boom of China's HREs industry. In contrast, demand for Tb increased at a relatively modest compound annual growth rate of 18% in the same period. This resulted in a surplus of China's Tb supply, with the gap expanding from 20 tons/yr in 1990 to 200 tons/yr in 2006. However, during the period from 2006 to 2018, China's Tb supply plummeted by up to 90%, while Tb demand soared. Consequently, China's Tb supply shifted from a surplus to a shortage in 2009, and by 2018 the official supply of Tb could only satisfy half of the demand.

### 3.2 Limited terbium supply under China's quota policy and HREs environmental regulations

**Fig. 2:**
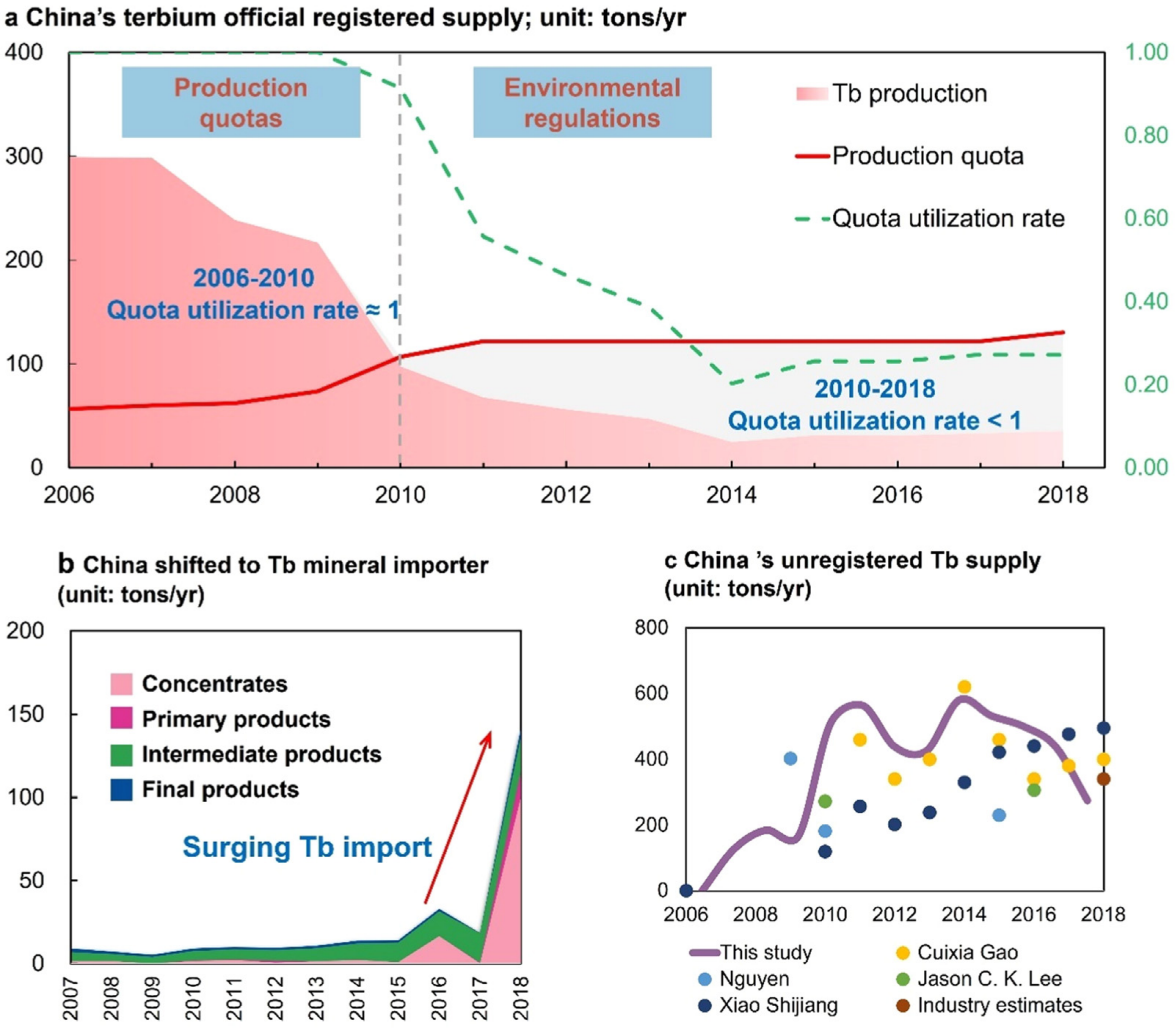
**China's terbium supply during 2006–2018.** (a) The official registered Tb supply under China's rare earth policies; (b) the evolution of China's Tb trade volume; (c) China's estimated unregistered supply.

Fig. 2a illustrates how China's Tb officially registered production evolved under the changes in China's rare earth production policies during 2006–2018. We have identified two distinct stages, with production quota policy and environmental regulations as the main reasons for the production variation in the respective stage.

In the initial stage spanning 2006–2010, China's officially registered Tb production notably declined from 300 tons/yr to 100 tons/yr, largely due to the influence of China's rare earth production quota policy. In response to resource depletion and environmental damage challenges posed by excessive exploitation and mining of HREs, the Chinese government implemented rare earth production quota policy in 2006. This policy guided the allowable amount of rare earth concentrate production quotas. Our results revealed a substantial gap between the actual demand and quotas, with 2006 Tb production quota of only 80 tons/yr, significantly lower than the officially registered production of 300 tons/yr. This disparity placed significant pressure on China's officially registered Tb production, forcing a rapid decline. Over time, however, the rare earth production quota was gradually relaxed. By 2010, Tb officially registered production in China had dropped to 100 tons/yr, nearly aligning with the quota for that year. In summary, the rare earth quota policy played a significant role in reducing Tb production in China during this stage.

In the second stage during 2010–2018, China's officially registered Tb production continued to decrease from 100 to 30 tons/yr, with the quota utilization rate for Tb plummeting to only 25% by 2018. This decline was primarily attributed to the enforcement of stricter environmental regulations within the HREs mining sector. In 2011, China enacted the first industrial standards for air and water pollution within the rare earth industry, which effectively prevented the approval of outdated mining techniques. In 2012, China further released entry conditions for the rare earth industry, explicitly prohibiting the use of outdated processes for HREs production (i.e., heap leaching and pool leaching techniques). Moreover, China conducted routine investigations and imposed crackdowns on HREs production activities that failed to comply with environmental regulations almost every year since 2010. In this context, nearly 90% of China's HREs production companies that failed to meet these stringent standards were compelled to halt their mining operations [17]. As a direct outcome, China's Tb officially registered production dropped from 100 tons/yr to 30 tons/yr. Meanwhile, China's quota relaxed to 150 tons/yr, indicating that the quota utilization rate for Tb dropping to only 25% by 2018. Such a result demonstrated that environmental issues at HREs mining activities, rather than China's production quota, have been the primary constraint on Tb supply in this stage.

Amidst the rapid decline in officially registered production, China's Tb supply heavily relied on imports and unregistered sources (including "illegal" mining, "illegal" imports, statistical discrepancies, etc.). Fig. 2b illustrates the substantial increase in China's Tb imports, peaking at 100 tons/yr in 2018 (in the same year, domestic official registered production was only 30 tons/yr). These imports primarily originated from Southeast Asian countries, as depicted in Fig. 1b. Additionally, China probably received significant amounts of unregistered Tb supply. Here we present our estimate of China's unregistered Tb supply, as well as results from existing literature [1,[38], [39], [40] and industrial estimates, as shown in Fig. 2c. The results show that since the implementation of the quota policy in 2006, China's unregistered Tb supply steadily rose from 150 tons/yr in 2007 to a peak of 500–600 tons/yr in 2011. In response to the Chinese government's stringent rectification and cracking efforts, the unregistered supply of Tb decreased to 300–400 tons/yr by 2018. In summary, China sourced a total of more than 5000 tons of Tb through unregistered routes in the studied period, surpassing its official registered supply of 3000 tons. It's important to note that these unregistered mining activities were often associated with small-scale workshops, outdated techniques, and caused severe environmental damage [41].

### 3.3 China's booming terbium demand

**Fig. 3:**
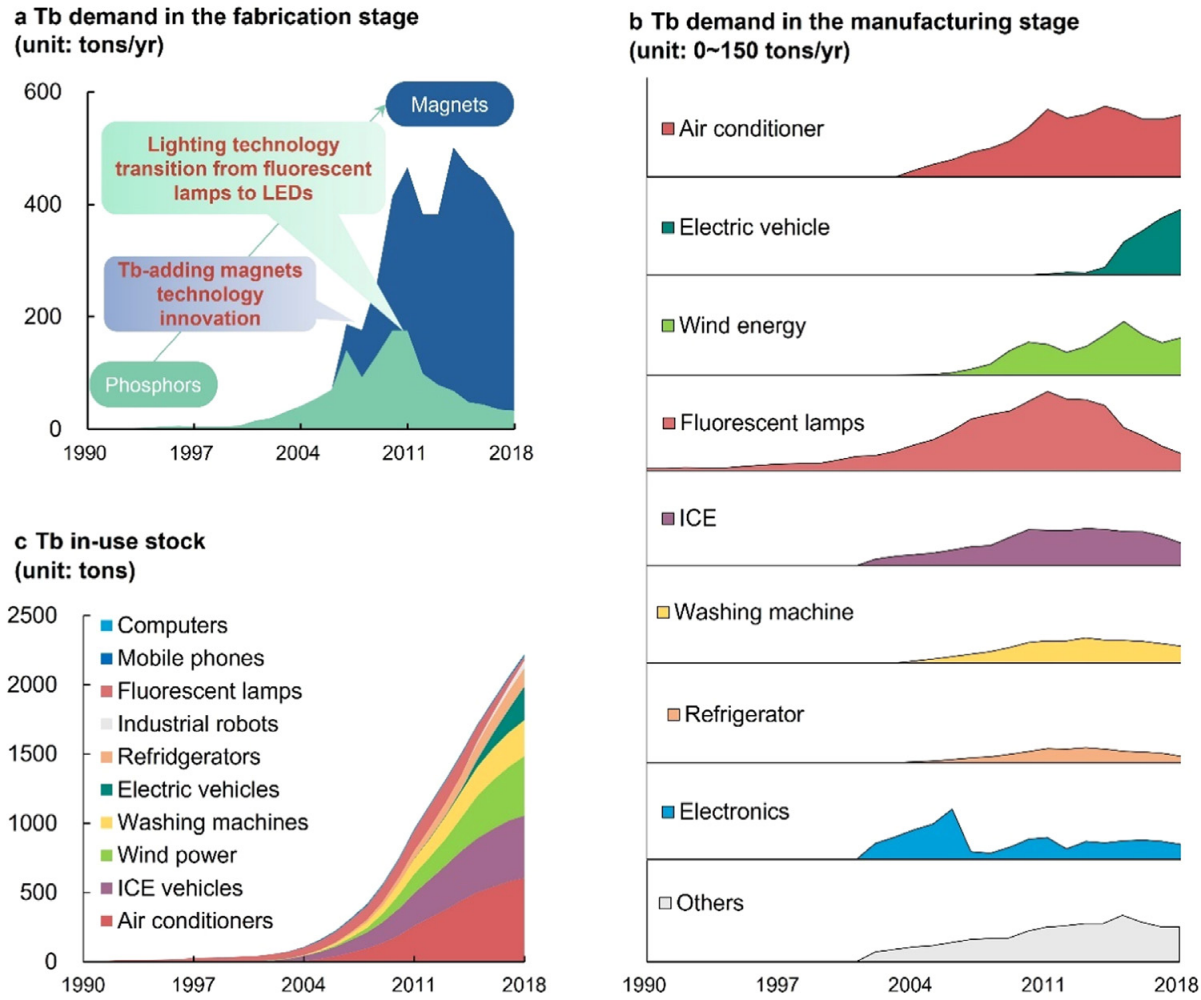
**Historical terbium demand in China during 1990–2018.** (a) Tb demand in the fabrication stage; (b) Tb demand in the manufacturing stage; (c) Tb in-use stock.

Fig. 3a shows that the demand for Tb boomed with a shift of major applications from tricolor phosphors to Nd-Fe-B permanent magnets, which is primarily driven by technological innovations. China's Tb demand increased from 10 tons/yr in 2000 to 350 tons/yr in 2018. Initially, Tb was mainly used in the lighting sector, leading to a steady 18% annual growth from 1990 to 2006. As a result, the grouth rate of Tb supply outpaced its demand , creating a supply surplus. However, a technology breakthrough, Tb-added Nd-Fe-B magnets, caused a 4-fold increase in Tb demand from 2006 to 2010. The rapid demand growth decreased the Tb surplus and eventually caused a shortage. Peak demand occurred in 2010 and 2015, after which technology evolved. In 2010, the lighting application shifted to light-emitting diodes (LEDs), causing a shrinking by about 80% in Tb demand in the lighting sector during 2010–2018. Additionally, the application of HRE reduction technology led to a 25% reduction in the Tb demand from Nd-Fe-B magnets during 2015–2018.

Similarly, Fig. 3b shows a rapid increase in final Tb demands with a marked change in application composition. Traditional applications, especially fluorescent lamps, constituted the vast majority (nearly 90%) of total Tb demand during 1990–2006. However, since 2010, emerging low-carbon applications, including electric vehicles, wind turbines, and energy-efficient home appliances, have dominated Tb demand. By 2018, these low-carbon applications made up about 80% of the total Tb demand. Tb in-use stocks increased continuously with an annual growth rate of 18% from 1990 to 2006 and 22% from 2007 to 2018, and finally reached approximately 2000 tons in 2018 (Fig. 3c). The share of traditional applications in total Tb stocks was at 90% in the 1990s but decreased since 2010. In contrast, the share of home appliances increased and constituted 40% of total Tb stocks in 2010.

### 3.4 Growing shortage risk and mitigation strategies

Here we project the future trends of the demand for Tb under the baseline scenario, the stated policies scenario (STEPS) scenario, and the Net Zero scenario. To assess Tb shortages, these demand projections are further compared with the assumed constant Tb supply since 2018 (excluding unregistered sources). Furthermore, this study further proposes four supply strategy scenarios and evaluates their potential to alleviate Tb shortages. The first scenario maximizes the utilization of released production quotas, while the second goes a step further by relaxing these quotas. It's important to note that the feasibility of the two scenarios depends on advancements in green mining techniques. The third and fourth scenarios explore the extensive utilization of recycling streams from end-of-life products and imports as significant feedstock sources, respectively.

**Fig. 4:**
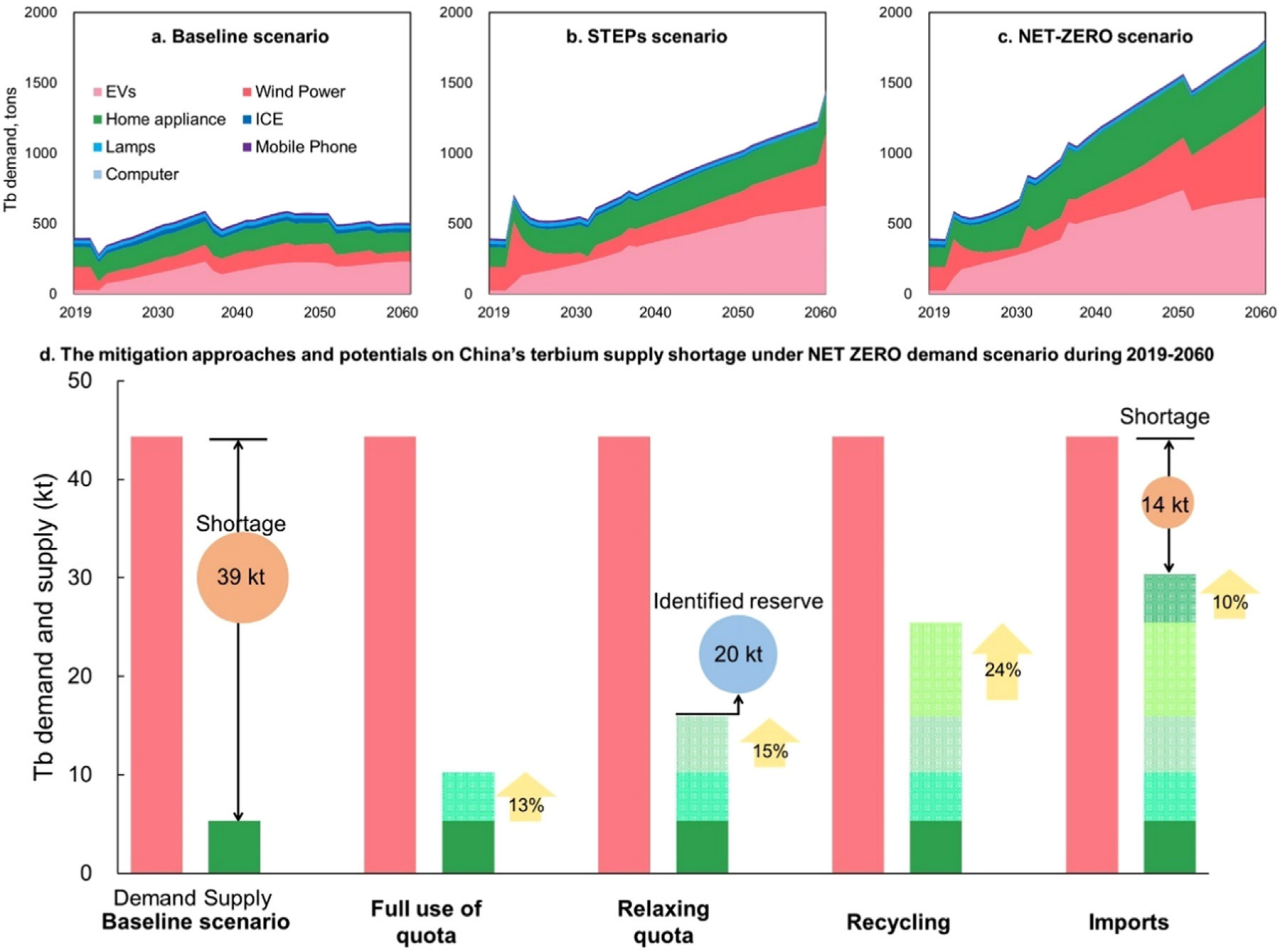
**Perspective terbium demand in China during 2020–2060 in the baseline scenario (a), STEPS scenario (b), the NET-ZERO scenario (c), and shortage as well as mitigation strategies (d)**.

Fig. 4a-c shows a divergent China's future Tb demand under three distinct scenarios. The baseline scenario suggests a modest 1.2-fold increase in demand over the next four decades, reflecting a conservative outlook on the low-carbon transition. In contrast, the more aggressive STEPS scenario predicts a 4-fold surge, while the most ambitious Net-Zero scenario anticipates a 5-fold increase in Tb demand. In terms of the driving forces, EVs, wind turbines, and home appliances dominate Tb demand in all scenarios, collectively accounting for over 95% of the total Tb demand throughout the study period. Among these, EVs emerge as the primary driver, comprising 39%–52% of Tb total demand under the three scenarios, followed by wind turbines (16%–37%) and home appliances (18%–31%).

Our projections indicate a cumulative Tb demand ranging from 20,500 to 44,300 tons between 2019 and 2060 under the three demand scenarios. In stark contrast, China's current Tb reserve stands at approximately 20,000 tons, failing drastically short of meeting the immense demand, even if the entire identified reserve is mined. Furthermore, the existing Tb supply is severely limited, totaling only 5300 tons, which can fulfill just 26%, 16%, and 12% of the projected demand under the three scenarios. Consequently, there looms a huge substantial Tb supply shortage, ranging from 15,100 to 39,000 tons, posing a significant challenge for meeting the future demand.

Fig. 4d illustrates the mitigation potential of supply strategies under the Net Zero demand scenario. Results indicate that if China's quota could be fully utilized, Tb shortage is expected to decrease by 13% in the Net Zero demand scenario, 17% in the STEPS scenario, and a significant 26% in the baseline scenario. Assuming China's Tb production quota could soon reach its historical production peak (300 tons in 2006) and remain constant, an additional 15%, 20%, and 38% shortage could be alleviated under three scenarios. In summary, overcoming the environmental constraints related to HREs mining could contribute significantly, ranging from 27% to 70%, towards mitigating future Tb shortages across various demand scenarios.

Notably, as the HREs ores are constantly extracted from the lithosphere and further transferred into the final products, the Tb in-use stock accumulates. Consequently, the potential of Tb recycling from waste derived from this stock becomes substantial. Our finding indicates that the waste flow could reach a total of 16,600 tons throughout 2019–2060, equivalent to approximately 2/3 of Tb reserve. The actual recycling amount depends on the recycling rate, and here we assume such a rate from 4% to 80% (considering the loss of use stage, achieving a 100% recycling rate proves challenging). As a result, the recycling measures have the potential to provide 9500 tons Tb, alleviating shortage by 36%, 33%, and 24% under the three demand scenarios. Moreover, the supply shortage can be reduced by 22%, 14%, and 1% under different import scenarios.

## 4 Discussion

In the realm of HREs supply, China's quota policy and political landscape have often been perceived as constraints by some nations [42,43]. This study sheds light on a shift in the dominant factors influencing China's HREs supply from quota limitations to stringent environmental regulations. Results show that China's rare earth quota policy did lead to a reduction in officially registered HREs production during a limited period of 2006–2010. However, a pivotal change occurred when China progressively relaxed its HREs quota, surpassing officially registered production levels since 2010. Surprisingly, by 2018, only 25% of China's HRE production quota was utilized. This study underscores the constraint shifted to a conflict between the present mining techniques and the stringent environmental regulations. These findings highlight an urgent need to take mining environmental constraints, rather than political concerns, as a new dimension in the supply risk assessment framework for critical HREs. This shift in perspective has implications, urging a transition on HREs from geopolitical competition factors to innovative green supply solutions.

Historically, the high environmental impacts from mining have been disrupting the global rare earth supply frequently. The United States Mountain Pass and Australia's Lynas Corporation's processing plant located in Malaysia both had to close their operations in 1998 and 2002, respectively, mainly due to environmental pollutions [44,16]. As for China, the current three major types of HREs mining techniques could lead to diverse environmental damages regarding their specific processes and related chemicals. The first, the pond leaching technique, involves excavating topsoil and removing them into an elsewhere tank, and spraying them with NaCl-H₂C₂O₄ chemicals, resulting in severe surface vegetation destruction, soil erosion, and severe pollution of groundwater. The second, heap leaching technique, places the minerals in a mound and soaks them with (NH₄)₂SO₄-H₂C₂O₄ chemicals, which also leads to vegetation degradation and soil erosion. The third that has been widely adopted, namely in-situ leaching techniques, pumps (NH₄)₂SO₄-NH_4_HCO_3_ chemicals to the earth directly, produced large amounts of ammonium nitrogen, heavy metal and other pollutants. Hence, the environmental impacts of HREs mining should raise global attention.

According to our findings, we put forth the following policy recommendations for the advancement of China's HREs industry.

First and foremost, while some initiatives have been conducted, the government needs to continue to encourage and support HREs mining enterprises and affiliated scientific research institutes to engage in green mining technological innovation. For instance, a dedicated research group had long focused on improving HREs mining techniques, proposing an environmental engineering model that involves meticulous monitoring and assessment of environmental impacts at each process [45]. Additionally, another Chinese research team has suggested the substitution of ammonium sulfate with magnesium sulfate to mitigate ammonia nitrogen pollution [46]. Furthermore, a recent study has introduced a highly efficient technology utilizing electrokinetic processes, demonstrating a significant reduction in the impacts of HREs mining in tests conducted from bench-scale to a small pilot [47]. Despite these advancements, these environmentally friendly mining technologies have not yet been adopted on a large scale. To promote the widespread adoption of eco-friendly mining techniques and, consequently ensure the supply security of terbium, governments must increase their investments in technological innovation and promotion.

Second, it is essential to promote the establishment of HREs recycling systems to alleviate the pressure on primary resources. Recycling HREs from end-of-life products is identified as a pivotal strategy to mitigate supply pressure and curb illegal mining activities [48]. Our results show that in-use Tb stock accounted for 13% of China's total Tb mineral reserves in 2018, a proportion expected to rise to 80% by 2060 (assuming no new resources are discovered during this period). Notably, we find the annual decommissioning amount of Tb, though currently small, is projected to sharply increase, reaching 80% of Tb demand in 2060. However, end-of-life Tb recycling faces significant challenges, urging government coordination, strategic planning, and rule-setting. First, HREs are intricately distributed within functional components, making extraction substantially more complex [49]. Addressing this challenge requires governments and leading companies to take the initiative in establishing marking and traceability systems for HREs. Moreover, the chemical similarities among HREs pose another hurdle in their separation [50], emphasizing the need for collaborative efforts between industries and academia to develop efficient separation technologies and establish demonstration plants. Additionally, it is crucial to consider the cost of Tb recycling. Government subsidies may be necessary to incentivize and facilitate the recycling process effectively.

Last, we strongly recommend intensifying international cooperation within the HREs industry. Despite being a major global producer of HREs, China is expected to grapple with severe shortages hindering its domestic sustainable transition. Our research indicates that importing HREs mineral products, even at the 2018 levels, could potentially alleviate 10%–15% of the Tb shortage in China. Moreover, mining prospecting operations have been conducted worldwide by various countries, leading to significant discoveries of HREs. For instance, substantial reserves have been discovered in foreign countries such as Greenland and Turkey. However, environmental concerns associated with mining also have constraints on HREs production on a global scale. For example, Greenland is drafting legislation to address environmental issues caused by mining activities, which could halt the development of the Kuannersuit mine, one of the world's largest HREs deposits [51]. Considering these challenges, we emphasize the urgent need for global initiatives in mineral exploration, innovation in green mining technologies, and expansion of mineral production.

This study involves an evaluation of unregistered terbium supply data, hence, necessitating validation and uncertainty assessment. In Fig. 2c, we present China's unregistered Tb supply results from our study, alongside estimates from various existing literature and industrial estimates. Results show that our estimates are, on average, 10%–30% higher overall than those from the literature and industry. While both our study and previous literature studies utilize upstream and downstream material balances for estimation, they differ in estimation boundaries, leading to divergent results. Specifically, the literature and industrial estimates only considered the production-consumption imbalance within China [39], whereas our research considers terbium consumption in major foreign markets, such as Japan and the EU. Given the assumption that China primarily supplies global terbium, the higher results observed in our study compared to the literature's findings can be attributed to this broader perspective. Moreover, there is considerable uncertainty surrounding the import scenario. Furthermore, a multitude of factors, encompassing geopolitics, trade constraints, export prohibitions, and environmental regulations, possess the capacity to curtail global rare-earth trade. As a result, this could lead to a decline in imports, consequently affecting China's supply and demand landscape.

Although MFA can help to trace the flows of Tb along its value chain, the influence of price and other market mechanisms on its supply risks and demand changes should also be evaluated, in which the MFA can serve as one solid foundation for such analysis. Hence, in the future, it will be imperative to delve into the factors that influence terbium prices and the market, considering market dynamics, demand-supply balances, and other external forces, for a more comprehensive analysis.

## 5 Conclusion

In conclusion, this study coupled dynamic material flow analysis with scenario analysis to uncover the cloudy bottlenecks of China's terbium supply chain. Our results revealed that environmental issues (rather than China's rare earth quota policy) have been the primary constraint on terbium supply since 2010, given that only 25% of China's terbium production quota was utilized in 2018. The findings underscore the urgent need for innovative green mining technologies to ensure a sustainable supply of Tb and other HREs. Our results also show that addressing environmental constraints related to HREs mining could mitigate 27%-70% of future Tb shortages across various scenarios. Hence, this study called for global attention on critical metals to shift from geopolitical competition to green supply, which is essential for securing the future supply of heavy rare earths.

## CRediT authorship contribution statement

P.W. and W.-Q.C. supervised the project. W.C. wrote the manuscript. F.-R.M, Alexandra, and Q-C.W contributed significantly to the final writing of this article. All authors approved the final manuscript.

## Declaration of competing interest

The authors declare that they have no conflicts of interest in this work.

## Appendix Supplementary materials

Image, application 1

## References

[1] Lee J.C.K., Wen Z (2018). Pathways for greening the supply of rare earth elements in China. Nat. Sustain..

[2] Zhou B., Li Z., Zhao Y. (2016). Rare Earth Elements supply vs. clean energy technologies: New problems to be solve. Gospod. Surowcami Miner..

[3] Humphries M. (2010).

[4] W.M. Morrison, Rachel Y. Tang, China's rare earth industry and export regime: Economic and trade implications for the United States. (2012) https://www.semanticscholar.org/paper/China's-Rare-Earth-Industry-and-Export-Regime%3A-and-Morrison-Tang/c6f12db2243c8e96db070ffc3c0c03af0e163e36.

[5] Sprecher B., Daigo I., Murakami S. (2015). Framework for resilience in material supply chains, with a case study from the 2010 rare earth crisis. Environ. Sci. Technol..

[6] Riddle M.E., Tatara E., Olson C. (2021). Agent-based modeling of supply disruptions in the global rare earths market. Resour. Conserv. Recycl..

[7] United States Department of Energy. (2010). https://www.energy.gov/sites/prod/files/edg/news/documents/criticalmaterialsstrategy.pdf.

[8] Nassar N.T. (2021).

[9] Nassar N.T., Du X., Graedel T.E (2015). Criticality of the rare earth elements. J. Ind. Ecol..

[10] European Commission. (2020). https://op.europa.eu/en/publication-detail/-/publication/c0d5292a-ee54-11ea-991b-01aa75ed71a1/language-en.

[11] European Commission. (2017).

[12] European Commission. (2014). https://rmis.jrc.ec.europa.eu/uploads/crm-report-on-critical-raw-materials_en.pdf.

[13] Hatayama H., Tahara K (2015). Criticality assessment of metals for japan's resource strategy. Mater. Trans..

[14] Australian Government. *Australia's critical minerals strategy*. (2019) https://www.industry.gov.au/news/australias-critical-minerals-strategy-released.

[15] Graedel T.E., Harper E.M., Nassar N.T. (2015). Criticality of metals and metalloids. Proc. Natl. Acad. Sci. U.S.A..

[16] Law Y.H. (2019). Politics could upend global trade in rare earth elements. Science.

[17] Shen Y., Moomy R., Eggert R.G. (2020). China's public policies toward rare earths, 1975–2018. Miner. Econ..

[18] The White House. Presidential executive order on assessing and strengthening the manufacturing and defense industrial base and supply chain resiliency of the United States. (2017) https://trumpwhitehouse.archives.gov/presidential-actions/presidential-executive-order-assessing-strengthening-manufacturing-defense-industrial-base-supply-chain-resiliency-united-states/.

[19] The White House. A federal strategy to ensure secure and reliable supplies of critical minerals. https://www.federalregister.gov/documents/2017/12/26/2017-27899/a-federal-strategy-to-ensure-secure-and-reliable-supplies-of-critical-minerals (2017).

[20] The White House. Executive order on addressing the threat to the domestic supply chain from reliance on critical minerals from foreign adversaries. (2020) https://trumpwhitehouse.archives.gov/presidential-actions/executive-order-addressing-threat-domestic-supply-chain-reliance-critical-minerals-foreign-adversaries/.

[21] Graedel T.E. (2019). Material flow analysis from origin to evolution. Environ. Sci. Technol..

[22] Graedel T.E., Harper E.M., Nassar N.T. (2013). On the materials basis of modern society. Proc. Natl. Acad. Sci..

[23] Nassar N.T., Graedel T.E., Harper E.M (2015). By-product metals are technologically essential but have problematic supply. Sci. Adv..

[24] Goldthau A., Westphal K., Bazilian M. (2019). How the energy transition will reshape geopolitics. Nature.

[25] NATIONAL SCIENCE AND TECHNOLOGY COUNCIL, (2018).

[26] National Research Council. Minerals, critical minerals, and the U.S. economy. (2007) https://nap.nationalacademies.org/resource/12034/critical_minerals_final.pdf.

[27] Energy, U. D. of Critical Materials Strategy (2010). https://energy.gov/sites/prod/files/edg/news/documents/Critical_Materials_Summary.pdf.

[28] European Commission. *Critical raw materials for strategic technologies and sectors in the EU: A foresight study*. 10.2873/865242 (2020) doi:10.2873/58081.

[29] Australian Government. (2020).

[30] Wang P., Chen L.Y., Ge J.P. (2019). Incorporating critical material cycles into metal-energy nexus of China's 2050 renewable transition. Appl. Energy.

[31] Ziemann S., Weil M., Schebek L (2012). Tracing the fate of lithium - the development of a material flow model. Resour. Conserv. Recycl..

[32] Severson M.H., Nguyen R.T., Ormerod J. (2023). An integrated supply chain analysis for cobalt and rare earth elements under global electrification and constrained resources. Resour. Conserv. Recycl..

[33] Reck B.K., Müller D.B., Rostkowski K. (2008). Anthropogenic nickel cycle: Insights into use, trade, and recycling. Environ. Sci. Technol..

[34] Licht C., Peiró L.T., Villalba G. (2015). Global substance flow analysis of gallium, germanium, and indium: Quantification of extraction, uses, and dissipative losses within their anthropogenic cycles. J. Ind. Ecol..

[35] Graedel T.E., Van Beers D., Bertram M. (2004). Multilevel cycle of anthropogenic copper. Environ. Sci. Technol..

[36] Wang Q.-C., Wang P., Qiu Y. (2020). Byproduct surplus: Lighting the depreciative Europium in China's rare earth boom. Environ. Sci. Technol..

[37] Wang Q., Chen W., Wang P. (2022). Illustrating the supply chain of dysprosium in China through material flow analysis. Resour. Conserv. Recycl..

[38] Xiao, S., Geng, Y., Pan, H., et al. Uncovering the key features of dysprosium flows and stocks in China. (2022) https://pubs.acs.org/doi/10.1021/acs.est.1c07724. 10.1021/acs.est.1c07724 35544346

[39] Gao C., Xu Y., Geng Y. (2022). Uncovering terbium metabolism in China: A dynamic material flow analysis. Resour. Policy.

[40] Nguyen R.T., Imholte D.D. (2016). China's rare earth supply chain: Illegal production, and response to new cerium demand. JOM.

[41] Liu H. (2016). http://www.chinawaterrisk.org.

[42] The White House. Remarks by President Biden at signing of an executive order on supply chains. (2021) https://bidenwhitehouse.archives.gov/briefing-room/speeches-remarks/2021/02/24/remarks-by-president-biden-at-signing-of-an-executive-order-on-supply-chains/.

[43] The White House. Building resilient supply chains, revitalizing American manufacturing, and fostering broad-based growth 100-day reviews under executive order 14017. (2021) https://bidenwhitehouse.archives.gov/wp-content/uploads/2021/06/100-day-supply-chain-review-report.pdf.

[44] USGS (2003). Mineral commodity summaries 2003 mineral commodity summaries 2003. Office.

[45] Li Y.-X., Zhang L., Zhou X.-M. (2010). Resource and environment protected exploitation model for lon-type rare earth deposit in southern of China (in Chinese). Rare Earth.

[46] Xiao Y.F., Huang X.W., Feng Z.Y. (2015). Progress in the green extraction technology for rare rarth from Ion − adsorption type rare rarths ore (in Chinese). Chin. Rare Earth.

[47] Wang G., Xu J., Ran L. (2023). A green and efficient technology to recover rare earth elements from weathering crusts. Nat. Sustain..

[48] Rademaker J.H., Kleijn R., Yang Y. (2013). Recycling as a strategy against rare earth element criticality: A systemic evaluation of the potential yield of NdFeB magnet recycling. Environ. Sci. Technol..

[49] B.K. Reck, T.E. Graedel. (2012). Challenges in metal recycling. Science.

[50] Balaram V. (2019). Rare earth elements: A review of applications, occurrence, exploration, analysis, recycling, and environmental impact. Geosci. Front..

[51] Gronholt-pedersen, J. Greenland prepares legislation to halt large rare-earth mine. https://www.reuters.com/business/environment/greenland-prepares-legislation-halt-large-rare-earth-mine-2021-09-17/(2021).

