# Supplementary material for: Reshaping heavy rare earth supply chains amidst China's stringent environmental regulations
Source: Fundam Res. 2024 Jan 26;5(2):505–13. doi: 10.1016/j.fmre.2023.11.019 (PMC11997559; doi:10.1016/j.fmre.2023.11.019)
Supplement: Supplementary file 1 [file mmc1.pdf]

Supporting Information for

Reshaping Heavy Rare Earth Supply Chains Amidst China's Stringent Environmental Regulations

Wei Chen,[1,2,7,] Peng Wang,[1,3,4,7,*], Fanran Meng[5], Alexandra Pehlken[6], Qiao-Chu Wang[1], and Wei-Qiang Chen,[1,2,4,*]

[1] Key Lab of Urban Environment and Health, Institute of Urban Environment, Chinese Academy of Sciences, 1799 Jimei Road, Xiamen, Fujian, 361021, China

[2] Department of Environmental Science and Engineering, University of Science and Technology of China, 230026 Hefei, China

[3] Ganjiang Innovation Academy, Chinese Academy of Science, Ganzhou, 341119, China

[4] University of Chinese Academy of Sciences, Beijing, 100049, China

[5] Department of Chemical & Biological Engineering, The University of Sheffield, Mappin Street, Sheffield, S1 3JD, United Kingdom

[6] Cascade Use Research Group, University of Oldenburg, 26111, Oldenburg, Germany

[7] These authors contributed equally

[*]Correspondence:

pwang@gia.cas.cn (P.W.)

wqchen@iue.ac.cn (W.-Q.C.)

Contents

1 Supply chain quantification methods 3
Figure S1. The framework of the terbium cycle in China 6
Table S1. Quantifications of flows for China's terbium cycle 7
Table S2. HS codes of Tb-containing commodities 17
Table S3. The lifetime of various final products 20
2 HRE production quota quantification methods 21
Table S4. Tb content in each province in China 21
Figure S2. HRE production quota in China 21
3 The HREEs mining technology 22
4 HRE demand projection quantification methods 23
Figure S3. EVs inflow in China through 2060 24
Figure S4. Wind turbine inflow in China through 2060 24
Figure S5. Terbium flows in China 25
Figure S6. Terbium official registered production in China 27
Figure S7. Intermediate products demand for Tb in China 28
Figure S8. The trade volume of terbium in China 29
Figure S9. End-of-life of terbium in China 30
Figure S10. Results of uncertainty analysis 31
Reference 32

1 Supply chain quantification methods

The balance between supply and demand of rare earth elements became a problem as soon as purified REEs started to be used in applications, as is said in recent literature. This study explored the overall Tb supply-demand balance issue by comparing China's Tb official registered production from ores to its domestic demand for the fabrication process.

1.1 Supply-demand balance. An important issue is to clarify the concept of terbium surpluses and shortages. This study compares China's terbium official registered production from ores to its domestic demand for the fabrication process. A terbium surplus occurred when the official registered production of terbium exceeds domestic demand in the fabrication process in China. In contrast, a terbium shortage means that domestic official registered mineral production can't meet its demand for the fabrication process. A series of published literature revealed that a supply shortage exists in the rare earth industry. Imbalance, either surpluses or shortages, can be calculated by the following equals:

$$F^{Surplus}(Tb,t) = F^{Supply}(Tb,t) - F^{Demand}(Tb,t) \quad (1)$$

$$F^{Shortage}(Tb,t) = F^{Demand}(Tb,t) - F^{Supply}(Tb,t) \quad (2)$$

where $F^{Surplus}(Tb,t)$ and $F^{Shortage}(Tb,t)$ indicate terbium surpluses and shortages in China in the year *t*, $F^{Supply}(Tb,t)$ indicates China's official registered mineral production of terbium in the year *t*, $F^{Demand}(Tb,t)$ indicates China's domestic demand for terbium in the year *t*.

The results of the terbium supply-demand balance can be found in Figure 3(c). China's terbium surpluses occurred during 1990-2008, while a terbium shortage has occurred since 2009.

1.2 China's official registered terbium production. The production of individual rare earth elements (e.g., terbium) is entirely determined by the availability of rare earth mines. Further, China's rare earth concentrate production is determined by the demand for several elements (i.e., neodymium and dysprosium). Besides, China's rare earth production is also affected by environmental regulations and production quotas. Hence, the officially registered production of terbium should be calculated by the Eq (3) and (4):

$$F^{Pro}(Tb,t) = \sum_1^k (F^{Pro}(REs,t) \times F^{Distribution}(REs,Tb)) \quad (3)$$

$$F^{Pro}(REs,t) = G(Quotas, Envir\ issues, Demand) \quad (4)$$

Where $F^{Pro}(Tb,t)$ indicates the official registered production of terbium in China in the year t, $F^{Pro}(REs,t)$ indicates the official registered production of rare earth concentrate in the year t, $F^{Distribution}(REs,Tb)$ indicates the distribution of terbium in the rare earth mines, k indicates China's major rare earth mining deposits (Bayan Obo mine in northern China, Bastnaesite mine in Sichuan and Shandong Province, ionic clays in southern China, and others). Eq (4) indicates that the production of China's rare earth is affected by the demand of downstream users, production quotas published by the Chinese government, and environmental regulations. The results of China's official registered terbium production can be found in Figure S2.

1.3 Domestic terbium surplus and export. Our results show that terbium surpluses occurred in China during 1990-2008. Hence, it's implied that the surpluses of terbium were needed to be either exported or stockpiled. However, we face a great challenge because neither data on export nor information about terbium stockpile is available or clear. On one hand, trade data on terbium refined products (i.e., Tb oxides, Tb metals) must be obtained via 8-digit HS code, which has been available since 2006 (the detailed information about HS

code of Tb-containing products along the life cycle is presented in Table S2). On the other hand, China has emphasized the stockpile issue of terbium since 2007. On this basis, this study made a feasible assumption that China's terbium surpluses during 1990-2006 were used to export to other nations.

1.4 Domestic terbium shortages and imports. Our results show that domestic terbium shortages have occurred in China since 2009. As mentioned before, the so-called "shortages" were calculated by comparing domestic official registered production in the mining stage to the demand for the fabrication process, while the trade (i.e., refined products export) was not considered. If we took the trade issue into account, China's terbium official registered supply couldn't meet its demand (including downstream consumption and export) since 2007, as shown in Figure S1. Importing terbium from other nations is helpful to solve the shortage. However, there is still an imbalance between China's terbium supply (including domestic mineral production and import) and its demand. Notably, lots of literature has found the imbalance of supply-demand for rare earth elements and indicated that a considerable amount of rare earth resources is from unregistered or undocumented mining activities[1–4]. And in this study, "shortage" was what we called the imbalance, as shown in Figure S1.

Figure S1. The framework of the terbium cycle in China

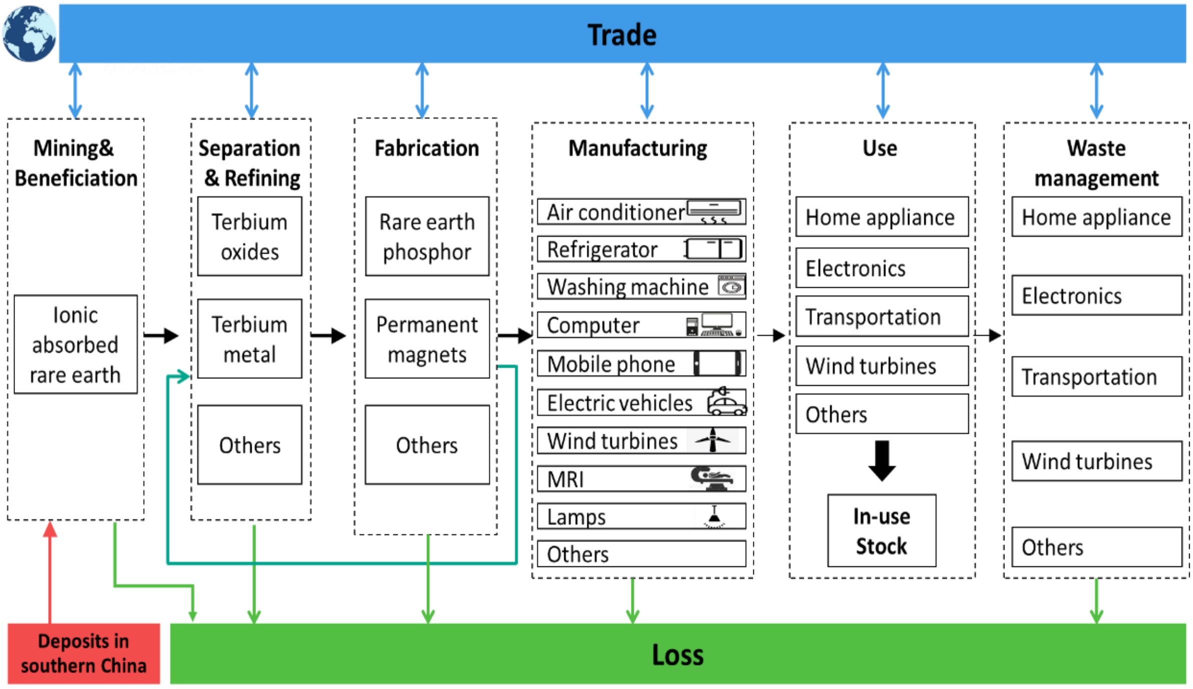

Table S1. Quantifications of flows for China's terbium cycle

Process	Symbol	Description	Equation	Data source
1.1 Bayan Obo **Mining & Concentration**	Inflow	Tb input to this process	Inflow=Outflow/RR	Mass balance
	Outflow	Tb primary products from this process	From official statistics (REO, Tb share: 0.01%)	[5]
	Loss	Tb loss from this process	Loss=Inflow-Outflow	Mass balance
	RR	Tb resource recovery rate of this process	10%	[6]
1.2 Bastnaesite **Mining & Concentration**	Inflow	Tb input to this process	Inflow=Outflow/RR	Mass balance
	Outflow	Tb primary products from this process	From official statistics (REO, Tb share: 0.09%)	[5]
	Loss	Tb loss from this process	Loss=Inflow-Outflow	Mass balance
	RR	Tb resource recovery rate of this process	65%	[6]
1.3 Ionic Clays **Mining & Concentration**	Inflow	Tb input to this process	Inflow=Outflow/RR	Mass balance
	Outflow	Tb primary products from this process	From official statistics (REO, Tb share: 0.77%)	[5]
	Loss	Tb loss from this process	Loss=Inflow-Outflow	Mass balance

Process	Symbol	Description	Equation	Data source
	RR	Tb resource recovery rate of this process	75%	[6]
1.4 Others **Mining & Concentration**	Inflow	Tb input to this process	Inflow=Outflow/RR	Mass balance
	Outflow	Tb primary products from this process	From official statistics (REO, Tb share: 0.55%)	[5]
	Loss	Tb loss from this process	Loss=Inflow-Outflow	Mass balance
	RR	Tb resource recovery rate of this process	50%	Average of all other three projects

Process	Symbol	Description	Equation	Data source
2.1 Bayan Obo **Smelting & Refining**	Inflow	Tb input to this process	Inflow=Outflow	Mass balance
	Outflow	Tb primary products from this process	Outflow=Inflow*RR	Mass balance
	Loss	Tb loss from this process	Loss=Inflow-Outflow	Mass balance
	RR	Tb resource recovery rate of this process	From official requirement (86%)	[6]
2.2 Bastnaesite **Smelting & Refining**	Inflow	Tb input to this process	Inflow=Outflow	Mass balance
	Outflow	Tb primary products from this process	Outflow=Inflow*RR	Mass balance
	Loss	Tb loss from this process	Loss=Inflow-Outflow	Mass balance
	RR	Tb resource recovery rate of this process	From official requirement (86%)	[6]
2.3 Ionic Clays **Smelting & Refining**	Inflow	Tb input to this process	Inflow=Outflow	Mass balance
	Outflow	Tb primary products from this process	Outflow=Inflow*RR	Mass balance
	Loss	Tb loss from this process	Loss=Inflow-Outflow	Mass balance
	RR	Tb resource recovery rate of this process	From official requirement (92%)	[6]
2.4 Others **Smelting & Refining**	Inflow	Tb input to this process	Inflow=Outflow	Mass balance
	Outflow	Tb primary products from this process	Outflow=Inflow*RR	Mass balance
	Loss	Tb loss from this process	Loss=Inflow-Outflow	Mass balance

Process	Symbol	Description	Equation	Data source
	RR	Tb resource recovery rate of this process	From official requirement (90%)	[6]

Process	Symbol	Description	Equation	Data source
3.1 Nd-Fe-B **Fabrication**	Inflow	Tb input to this process	Inflow=Outflow/RR	Mass balance
	Outflow	Tb functional material from this process	Outflow=Production*Penetration Rate (PR)*Content<br>Production of Nd-Fe-B<br>PR: 0%(2006); 25%(2018)<br>Content: 6%（2006）; 1%(2018）	From publication<br>[7,8]<br>[9–12]
	Loss	Tb loss from this process	Loss=Inflow-Outflow<br>Loss rate: 25%	Mass balance<br>[11]
	RR	The recycling rate of new scrap	Recycling rate: 90%	
3.2 Rare Earth Phosphor **Fabrication**	Inflow	Tb input to this process	Inflow=Outflow/RR	Mass balance
	Outflow	Tb functional material from this process	Outflow=Production*Penetration Rate*Content<br>Production of Phosphor<br>Penetration Rate: 100%<br>Tb phosphors production Content: 0.01-0.03 g Tb/g	From publication<br>[13–15]
	Loss	Tb loss from this process	Loss=Inflow-Outflow	Mass balance

125

Process	Symbol	Description	Equation	Data source
4.1 Wind turbines	Inflow	Tb input to this process	Inflow=Production *Market Share*Content	Chinese Wind Energy Association
	PR	Penetration Rate	PR: 2006(0%); 2016(33%)	[16]
	Tb content	Tb content	7 t/GW	
	Outflow	Tb output from this process	Outflow= Inflow-Export+ Import	
	Export	Tb export to rest of world	Trade volume * Tb content	UN Comtrade
	Import	Tb import from rest of world		
4.2 Electric vehicle (EV)	Inflow	Tb input to this process	Inflow=Production *Market Share* Magnet weight per unit * Composition of Tb	Direct calculation
	PR	Penetration Rate	100%	[17,18]
	Tb content	Magnet weight per unit	BEV: 75 g/unit	
		Composition of Tb	1%-6%	
	Outflow	Tb output from this process	Outflow= Inflow-Export+ Import	
	Export	Tb export to rest of world	Trade volume * Tb content	UN Comtrade
	Import	Tb import from rest of world		
4.3 Plug-in Hybrid Electric Vehicle (PHEV)	Inflow	Tb input to this process	Inflow=Production *Market Share* Magnet weight per unit * Composition of Tb	Direct calculation
	PR	Penetration Rate	100%	[17,18]
	Tb content	Magnet weight per unit	PHEV: 15 g/unit	
		Composition of Tb	1%-6%	
	Outflow	Tb output from this process	Outflow= Inflow-Export+ Import	
	Export	Tb export to rest of world	Trade volume * Tb content	UN Comtrade
	Import	Tb import from rest of world		
4.4 Passenge	Inflow	Tb input to this process	Inflow=Production *Market Share*	Direct calculation

Process	Symbol	Description	Equation	Data source
r vehicle (ICE)			Magnet weight per unit * Composition of Tb	
	PR	Penetration Rate	1990: 0%; 2018: 40%	
		Magnet weight per unit	ICE: 2.5 g/unit	[14]
	Tb content	Composition of Tb	1%-6%	
	Outflow	Tb output from this process	Outflow= Inflow-Export+ Import	
	Export	Tb export to rest of world		
	Import	Tb import from rest of world	Trade volume * Tb content	UN Comtrade
4.5 Air conditioner	Inflow	Tb input to this process	Inflow=Production* Market Share* Magnet weight per unit * Composition of Tb	National Bureau of Statistics
	PR	Penetration Rate	1990: 0%; 2018: 40%	[19]
		Magnet weight per unit	1.2 g/unit	[17,18,20]
	Tb content	Tb content	1%-6%	
	Outflow	Tb output from this process	Outflow= Inflow-Export+ Import	
	Export	Tb export to rest of world		
	Import	Tb import from rest of world	Trade volume * Tb content	UN Comtrade
4.6 Fridges	Inflow	Tb input to this process	Inflow=Production* Market Share* Magnet weight per unit * Composition of Tb	National Bureau of Statistics
	PR	Penetration Rate	1990: 0%; 2018: 40%	

Process	Symbol	Description	Equation	Data source
		Magnet weight per unit	0.5 g/unit	[14]
	Tb content	Tb content	1%-6%	
	Outflow	Tb output from this process	Outflow= Inflow-Export+ Import	
	Export	Tb export to rest of world	Trade volume * Tb content	UN Comtrade
	Import	Tb import from rest of world		
4.7 Washing machines	Inflow	Tb input to this process	Inflow=Production* Market Share* Magnet weight per unit * Composition of Tb	National Bureau of Statistics
	PR	Penetration Rate	1990: 0%; 2018: 40%	
		Magnet weight per unit	1.4 g/unit	[17,18,20]
	Tb content	Tb content	1%-6%	
	Outflow	Tb output from this process	Outflow= Inflow-Export+ Import	
	Export	Tb export to rest of world	Trade volume * Tb content	UN Comtrade
	Import	Tb import from rest of world		
4.8 Mobile phones	Inflow	Tb input to this process	Inflow=Production* Market Share* Magnet weight per unit * Composition of Tb	Ministry of Industry and Information Technology
	PR	Penetration Rate	100%	
		Magnet weight per unit	0.025 g/unit	[17,18,20]
	Tb content	Tb content	1%-6%	
	Outflow	Tb output from this process	Outflow= Inflow-Export+ Import	
	Export	Tb export to rest of world	Trade volume * Tb content	UN Comtrade

**Process**	**Symbol**	**Description**	**Equation**	**Data source**
	Import	Tb import from rest of world		
4.9 Personal Compute r	Inflow	Tb input to this process	Inflow=Production *Market Share* Magnet weight per unit * Composition of Tb	Ministry of Industry and Information Technology
	PR	Penetration Rate	100%	
		Magnet weight per unit	0.03 g/unit	[17,18,20]
	Tb content	Tb content	1%-6%	
	Outflow	Tb output from this process	Outflow= Inflow-Export+ Import	
	Export	Tb export to rest of world	Trade volume * Tb content	UN Comtrade
	Import	Tb import from rest of world		
4.10 Phospho rs lamp	Inflow	Tb input to this process	Inflow=Production *Market Share* Magnet weight per unit * Composition of Tb	Direct calculation
	PR	Penetration Rate	100%	
		Magnet weight per unit	0.01~2 g/unit	[17,18,20]
	Tb content	Tb content	1%-6%	
	Outflow	Tb output from this process	Outflow= Inflow-Export+ Import	
	Export	Tb export to rest of world	Trade volume * Tb content	UN Comtrade
	Import	Tb import fror of world		
4.11 MRI	Inflow (4.11)	Inflow=Production* Market Share* Magnet weight per	Inflow=Production *Market Share* Magnet weight per	Direct calculation

**Process**	**Symbol**	**Description**	**Equation**	**Data source**
		unit * Composition of Tb	unit * Composition of Tb	
	PR	Penetration Rate	100%	
		Magnet weight per unit	0.03 g/unit	14
	Tb content	Tb content	1%-6%	
	Outflow (4.11)	Tb output from this process	Outflow= Inflow-Export+ Import	
	Export	Tb export to rest of world		
	Import	Tb import from rest of world	Trade volume * Tb content	UN Comtrade
4.12 Elevator	Inflow (4.12)	Tb input to this process	Inflow=Production *Market Share* Magnet weight per unit * Composition of Tb	National Bureau of Statistics
	PR	Penetration Rate	100%	
		Magnet weight per unit	60 g/unit	14
	Tb content	Tb content	1%-6%	
	Outflow (4.12)	Tb output from this process	Outflow= Inflow-Export+ Import	
	Export	Tb export to rest of world		
	Import	Tb import from rest of world	Trade volume * Tb content	UN Comtrade

126

Table S2. HS codes of Tb-containing commodities

Process	Tb-containing commodities	HS Codes	Description
Mining Mineral products	Concentrates	25309020 28469048	Concentrates: export<br>Concentrates: import from the USA<br>Concentrates: import from Australia<br>Concentrates: import from Malaysia<br>Concentrates: import from Burma
Smelting & Refining Refined products	Primary products	28469016	Tb oxide
		28053013	Tb metal
		28469021	Tb chloride
		28469093	Other Tb compounds
Fabrication Immediate Products	Nd-Fe-B magnets	85051110 850511	Permanent magnets o rare-earth metals
	Lamp phosphors	320650	Rare-earth phosphors

Process	Tb-containing commodities	HS Codes	Description
Manufacturing End-uses	Wind turbines	853931<br>850231	Wind turbines
	EV	870380<br>871190	Motor cars and other motor vehicles, with an only electric moto for propulsion
	ICE	870333<br>870390	Motor cars and other motor vehicles principally designed for the transport of persons
	Air conditioner	841510	Separate window or wall air conditioners
	Fridges	841810<br>841821<br>841822<br>841829	Compressed household refrigerators
	Washing machines	845011<br>845012<br>845019<br>845020	Wave wheel type automatic washing machines; Drum type automatic washing machines; Other washing machines with centrifugal drye
	Mobile phones	852520<br>851712	Telephones for cellular networks "mobile telephones" or for other wireless networks, Products include: Smart Phone
	Personal Computer	847120<br>847130<br>850231	Data-processing machines, automatic, portable
	Phosphor lamp	853931	Discharge lamps, fluorescent, hot cathode Products

Process	Tb-containing commodities	HS Codes	Description
	Elevator	842810<br>842820<br>842831<br>842832<br>842833<br>842839	Lifts and skip hoists

Table S3. The lifetime of various final products

Final Products	Average Lifetime (years)	References
Wind turbines	20	[2,19]
EV	8	[2,19]
ICE	8	[2,19]
Air conditioner	13	[2,19]
Fridges	12	[2,19]
Washing machines	10	[2,19]
Mobile phones	5	[2,19]
Personal Computer	7	[2,19]
Phosphor lamp	3	[13]
Elevator	15	[2,19]

2 HRE production quota quantification methods

Table S4. Tb content in each province in China

Tb_4O_7	Inner Mongolia	Shandong	Sichuan	Jiangxi	Fujian	Guangdong	Hunan
w%	<0.01	0.14	0.08	0.68	0.74	0.70	0.97

Figure S2. HRE production quota in China

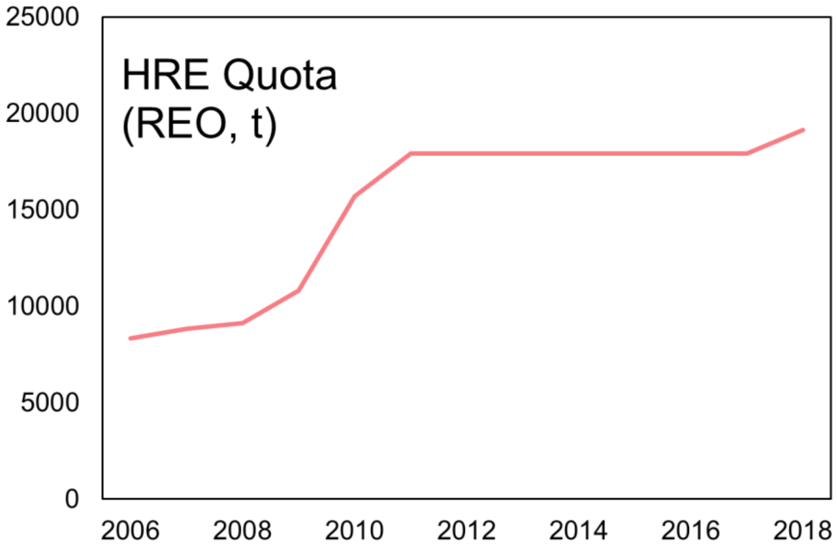

Source data: Shen[3]

3 The HREEs mining technology

There are three major methods for HREEs mining, and all of them lead to severe environmental issues.

The first, pool leaching technology, involves excavating topsoil, removing them into a different tank (causing severe surface vegetation destruction and soil erosion), and spraying them with NaCl-$H_2C_2O_4$ chemicals (the chemicals may leak to groundwater and affect entire waterways).

The second heap leaching technology places the minerals in a mound and soaks with $(NH_4)_2SO_4$-$H_2C_2O_4$ chemicals, which also leads to vegetation degradation and soil erosion.

The third in-situ leaching technology pumps $(NH_4)_2SO_4$-NH_4HCO_3 chemicals to the earth directly, which causes serious soil salinization, ammonia nitrogen pollution, and surface and groundwater contamination.

Hence, as a report shows, ammonia nitrogen levels in the Wojiang River (in Longnan, one of the main sources of HREEs in China) were about 300 times higher than the maximum allowed amount for China's Grade III Surface Water Standard in 2013, and in 2012 the drinking water of more than 30,000 people from Longnan was affected due to the mining activities of local HREEs.[21]

4 HRE demand projection quantification methods

Climate scenarios layer. This study follows the scenarios released by International Energy Agency, which basically include the baseline scenario, the National Determined Contributions (NDC) scenario, the Stated Policies Scenario (STEPs), the Announced Pledges Scenario (APS), and the NET ZERO scenario. Here we select the baseline scenario, the Stated Policies Scenario (STEPs), and the NET ZERO scenario in our scenario layer. In the baseline scenario, we assume the development trends of all applications remain as usual, suggesting that the current industries continue to operate under minimal policy intervention. The Stated Policies Scenario (STEPs) explores where the energy system might go and does not take for granted that governments will reach all announced goals. The NET ZERO scenario is the most ambitious scenario, pursuing the most rapidly expansion of low-carbon infrastructure.

Low-carbon infrastructure layer. According to the historical flows and stocks results, here we project the demand for Tb in various applications. For electric vehicles, here we combined the population, GDP, and vehicles stocks per capita to derive the vehicles stocks in the future, and then estimate the stock of electric vehicles through the electrification rate under different climate scenarios. Then, a stock-driven model was applied to transfer the stock to inflow annually. The results were shown in the Figure S3. Similarly, the result for new installation of wind turbine was shown in Figure S4. And note that the results for other applications were not listed separately, and can be seen in the Figure 4.

Figure S3. EVs inflow in China through 2060

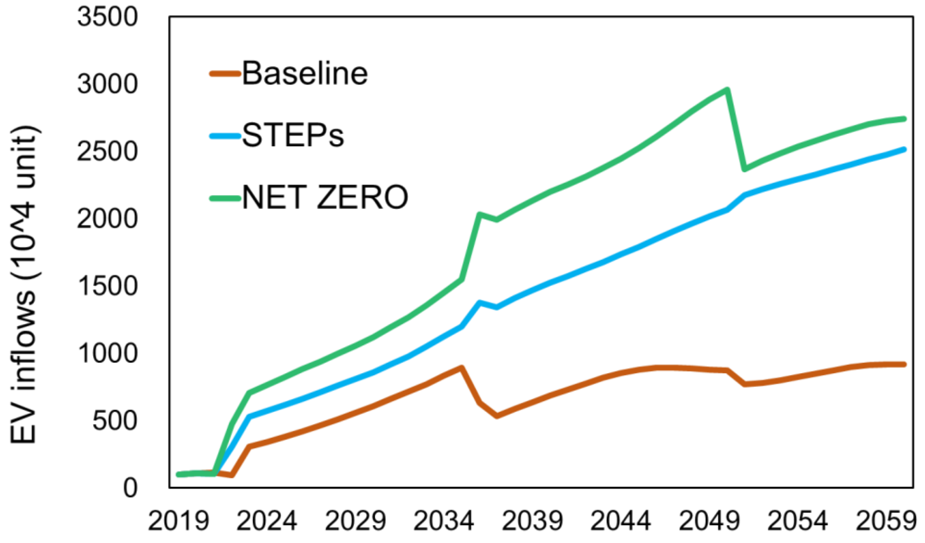

Figure S4. Wind turbine inflow in China through 2060

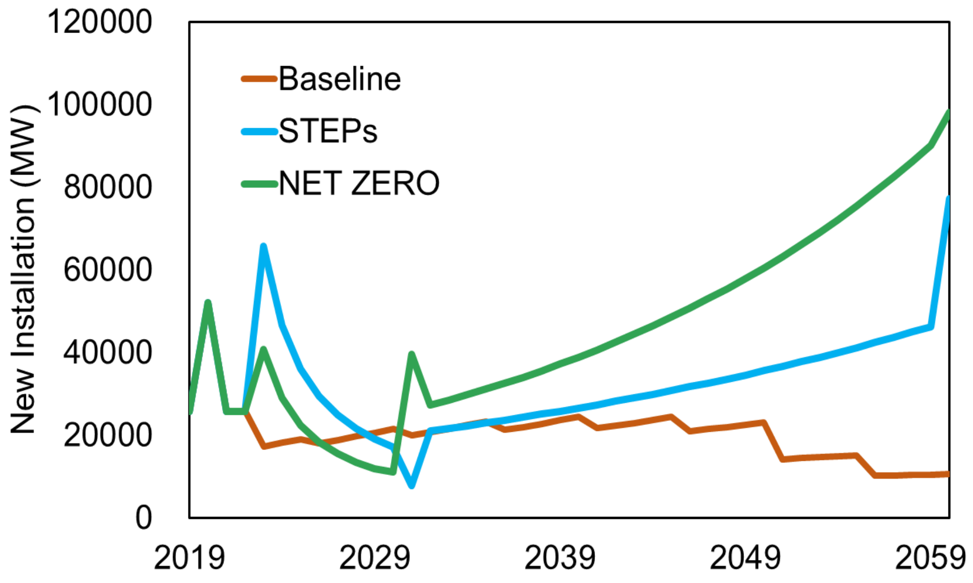

Figure S5. Terbium flows in China

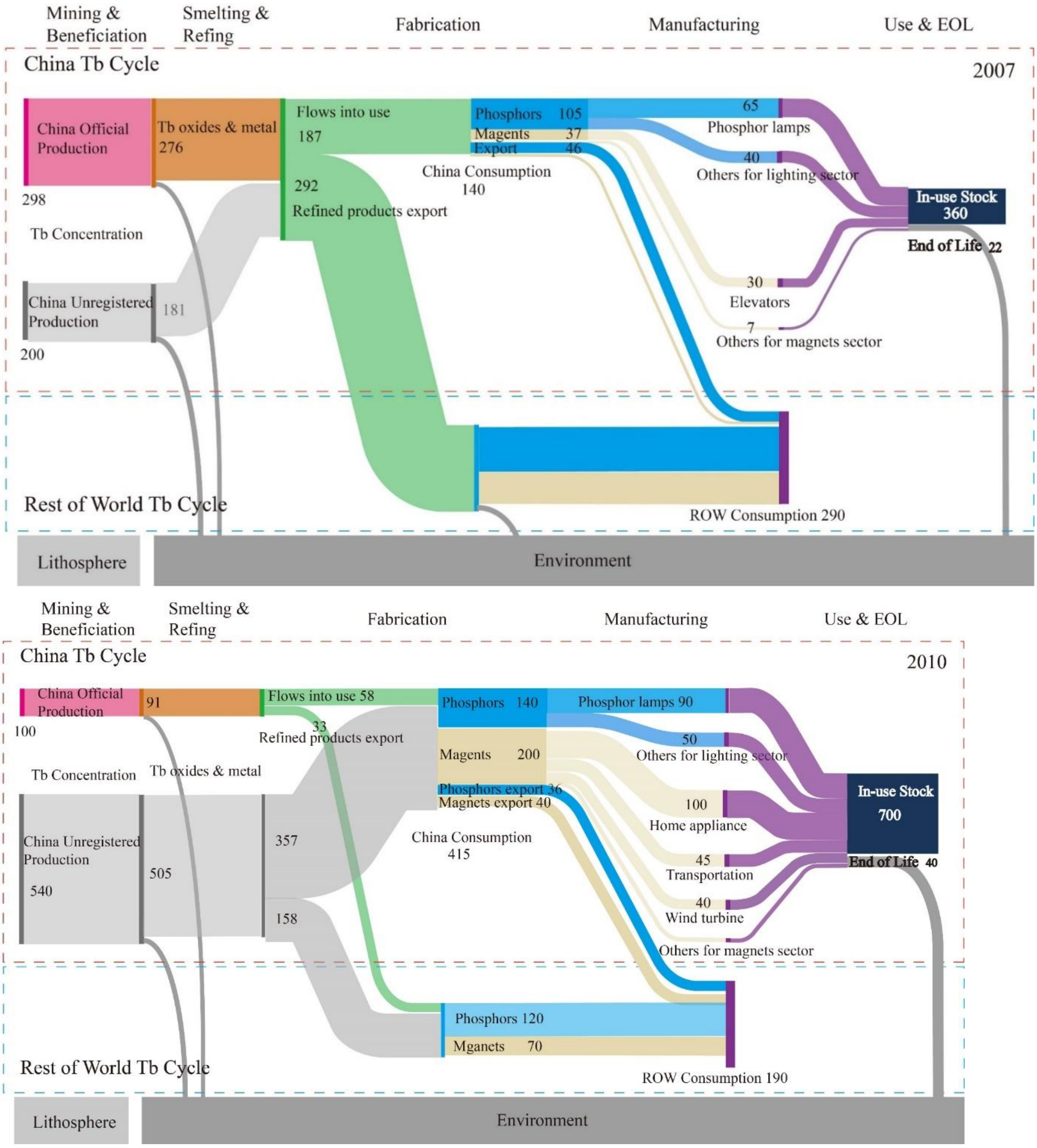

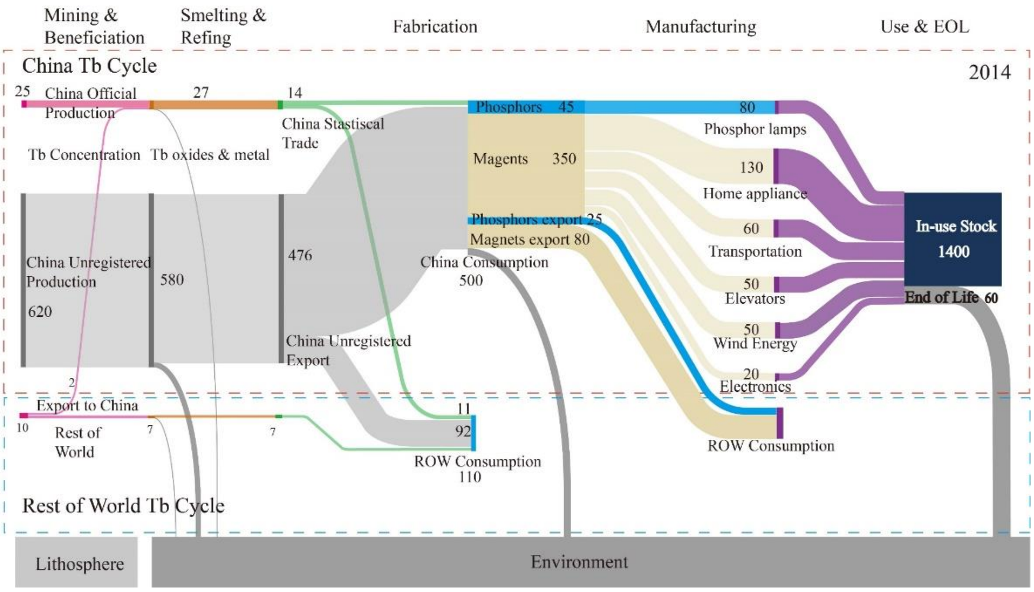
Mining & Beneficiation
Smelting & Refing
Fabrication
Manufacturing
Use & EOL
China Tb Cycle
2014
25
China Official Production
27
14
China Stastiscal Trade
Tb Concentration
Tb oxides & metal
Phosphors 45
Magents 350
80
Phosphor lamps
130
Home appliance
60
Transportation
50
Elevators
50
Wind Energy
20
Electronics
In-use Stock
1400
End of Life 60
Phosphors export 25
Magnets export 80
China Consumption
500
China Unregistered Production
620
580
476
China Unregistered Export
2
Export to China
10
Rest of World
7
7
11
92
ROW Consumption
110
ROW Consumption
Rest of World Tb Cycle
Lithosphere
Environment

Figure S6. Terbium official registered production in China

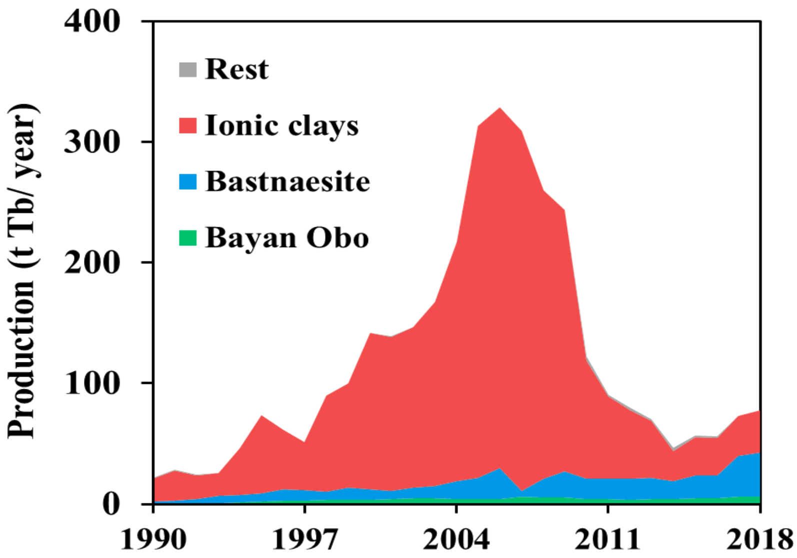

Figure S6. Terbium official registered production in China

Data from the reference literature[5]

Figure S7. Intermediate products demand for Tb in China

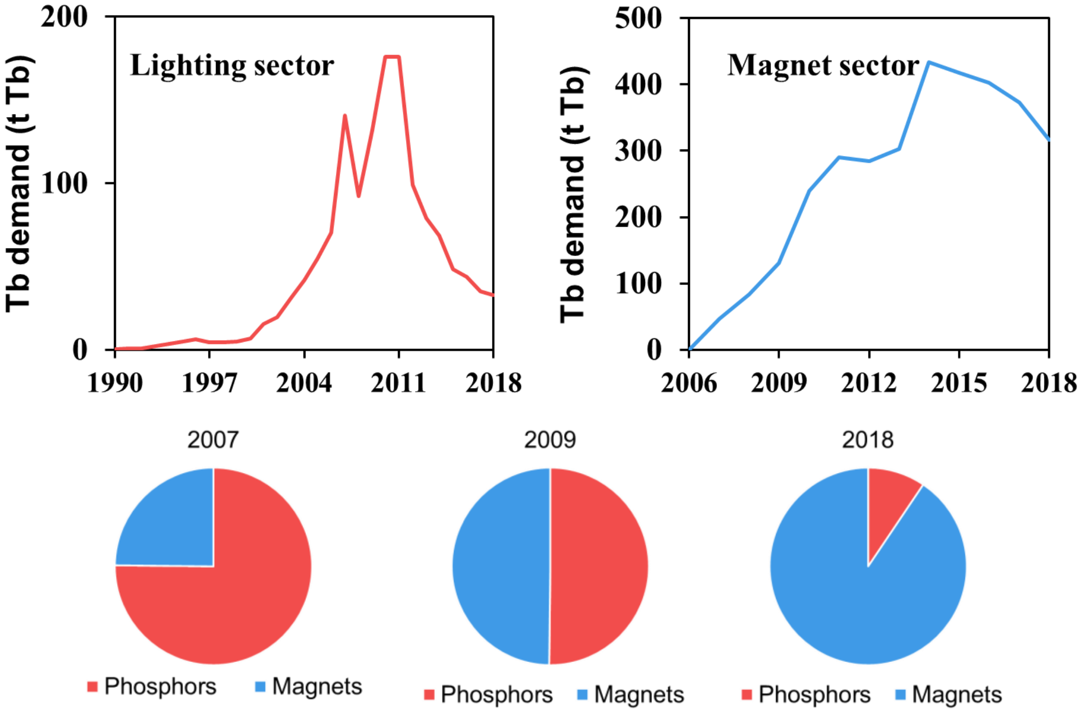

Figure S8. The trade volume of terbium in China

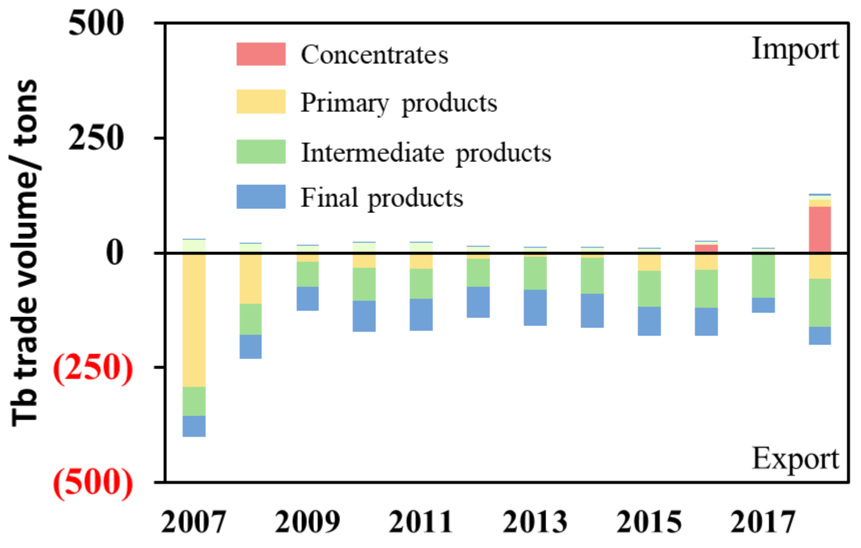

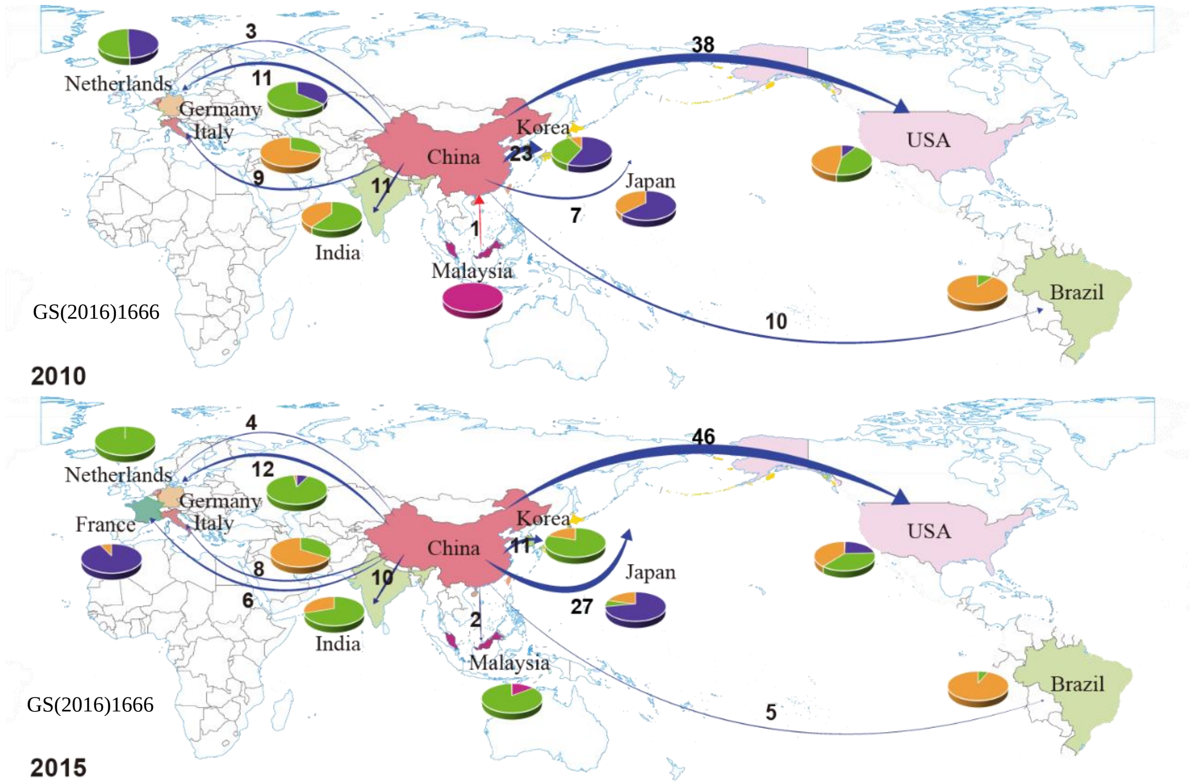

Figure S9. End-of-life of terbium in China

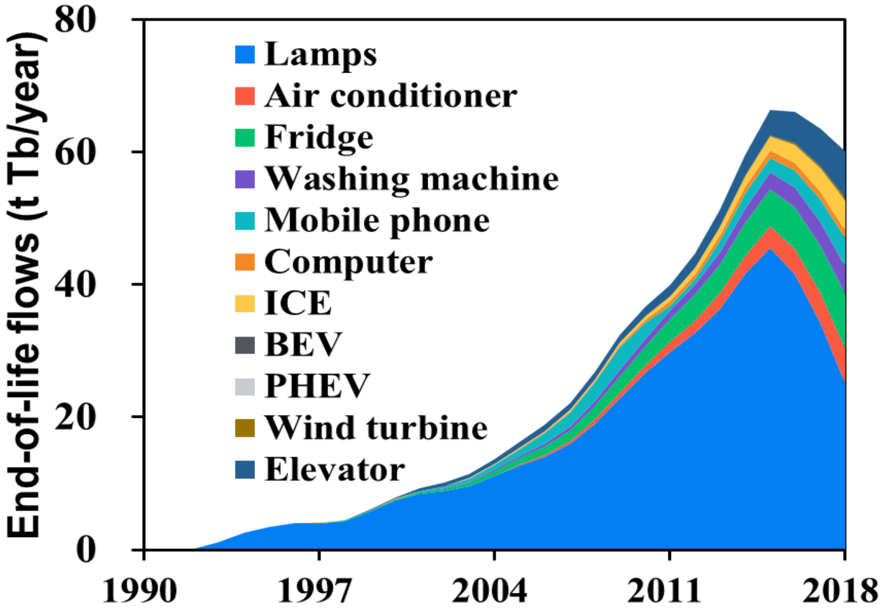

Figure S10. Results of uncertainty analysis

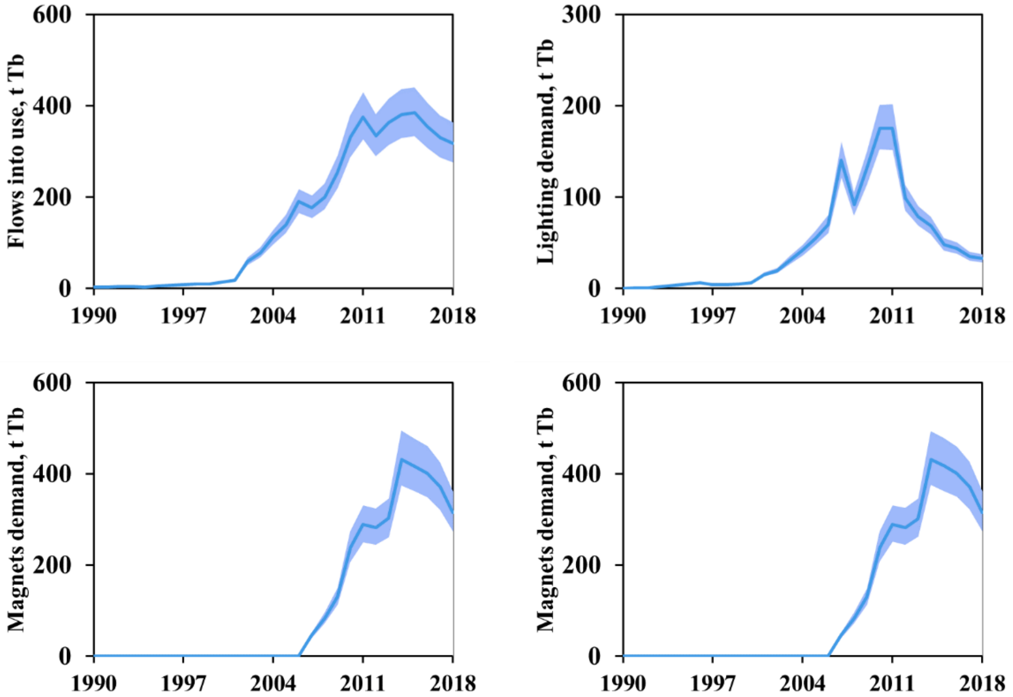

Figure S10. Uncertainty analysis for the results for the flows into use(a), demand for the lighting used when the content of Tb-containing products is different(b), demand for the magnet use when the content of Tb-containing products is different(c), demand for the magnet use when the market share of Tb-containing products is different(d).

Reference

1. Geng, J. *et al.* Static material flow analysis of neodymium in China. *J. Ind. Ecol.* 1–11 (2020).
2. Yao, T., Geng, Y., Sarkis, J., Xiao, S. & Gao, Z. Dynamic neodymium stocks and flows analysis in China. *Resour. Conserv. Recycl.* **174**, 105752 (2021).
3. Shen, Y., Moomy, R. & Eggert, R. G. China's public policies toward rare earths, 1975–2018. *Miner. Econ.* **33**, 127–151 (2020).
4. Nguyen, R. T. & Imholte, D. D. China's Rare Earth Supply Chain: Illegal Production, and Response to new Cerium Demand. *Jom* **68**, 1948–1956 (2016).
5. Society of China Rare Earth. *Chinese Society of Rare Earths Yearbook 2018 (in Chinese)*. (2018).
6. Ministry of Commerce, P. R. of C. Access conditions for the rare earth industry. http://www.mofcom.gov.cn/aarticle/b/g/201209/20120908362111.html (2012).
7. Liu Chengjun, L. L. Successful development of high-performance NdFeB permanent magnet materials (in Chinese). *Rare earth Inf.* (2002).
8. Chen Zhanheng. Rare earth industry and market in brief in 2018. rare earth information (in Chinese), 26–30 (2019).
9. Du, X. & Graedel, T. E. Global rare earth in-use stocks in NdFeB permanent magnets. *J. Ind. Ecol.* **15**, 836–843 (2011).
10. HONG, F. Rare Earth: Production, Trade and Demand. *J. Iron Steel Res. Int.* **13**, 33–38 (2006).
11. Sprecher, B. *et al.* Life cycle inventory of the production of rare earths and the subsequent production of NdFeB rare earth permanent magnets.

Environ. Sci. Technol. **48**, 3951–3958 (2014).

12. Guyonnet, D. *et al.* Material flow analysis applied to rare earth elements in Europe. *J. Clean. Prod.* **107**, 215–228 (2015).
13. Wang, Q.-C., Wang, P., Qiu, Y., Dai, T. & Chen, W.-Q. Byproduct Surplus: Lighting the Depreciative Europium in China's Rare Earth Boom. *Environ. Sci. Technol.* (2020).
14. Lee, I. S. & Kim, J. G. Industrial demand and integrated material flow of terbium in Korea. *Int. J. Precis. Eng. Manuf. - Green Technol.* **1**, 145–152 (2014).
15. Wu, Y., Yin, X., Zhang, Q., Wang, W. & Mu, X. The recycling of rare earths from waste tricolor phosphors in fluorescent lamps: A review of processes and technologies. *Resour. Conserv. Recycl.* **88**, 21–31 (2014).
16. Union, E. *Raw materials demand for wind and solar PV technologies in the transition towards a decarbonised energy system*. (2020).
17. Li, X. Y., Ge, J. P., Chen, W. Q. & Wang, P. Scenarios of rare earth elements demand driven by automotive electrification in China: 2018–2030. *Resour. Conserv. Recycl.* **145**, 322–331 (2019).
18. Securities, G. W. Focus on high-performance rare earth magnetic materials (in Chinese). http://newsrec.htsec.com:9087/Finchina_Data/rpt/FBTD/2019/2019-07/2019-07-31/4597922.pdf (2019).
19. Ciacci, L., Vassura, I., Cao, Z., Liu, G. & Passarini, F. Recovering the "new twin": Analysis of secondary neodymium sources and recycling potentials in Europe. *Resour. Conserv. Recycl.* **142**, 143–152 (2019).
20. Sekine, N., Daigo, I. & Goto, Y. Dynamic Substance Flow Analysis of Neodymium and Dysprosium Associated with Neodymium Magnets in Japan. *J. Ind. Ecol.* **21**, 356–367 (2017).

21. Liu, H. *Rare Earths: Shades of Grey - Can China Continue To Fuel Our Global Clean & Smart Future? China Water Risk* www.chinawaterrisk.org. (2016).
